# Applicability of F-specific bacteriophage subgroups, PMMoV and crAssphage as indicators of source specific fecal contamination and viral inactivation in rivers in Japan

**DOI:** 10.1371/journal.pone.0288454

**Published:** 2023-07-14

**Authors:** Yuno Meuchi, Miu Nakada, Keisuke Kuroda, Seiya Hanamoto, Akihiko Hata

**Affiliations:** 1 Graduate School of Engineering, Toyama Prefectural University, Imizu, Toyama, Japan; 2 Faculty of Engineering, Toyama Prefectural University, Imizu, Toyama, Japan; 3 Environment Preservation Center, Kanazawa University, Kanazawa, Ishikawa, Japan; Universidade Lisboa, Instituto superior Técnico, PORTUGAL

## Abstract

To date, several microbes have been proposed as potential source-specific indicators of fecal pollution. 16S ribosomal RNA gene markers of the *Bacteroidales* species are the most widely applied due to their predominance in the water environment and source specificity. F-specific bacteriophage (FPH) subgroups, especially FRNA phage genogroups, are also known as potential source-specific viral indicators. Since they can be quantified by both culture-based and molecular assays, they may also be useful as indicators for estimating viral inactivation in the environment. Pepper mild mottle virus (PMMoV) and crAssphage, which are frequently present in human feces, are also potentially useful as human-specific indicators of viral pollution. This study aimed to evaluate the applicability of FPH subgroups, PMMoV, and crAssphage as indicators of source-specific fecal contamination and viral inactivation using 108 surface water samples collected at five sites affected by municipal and pig farm wastewater. The host specificity of the FPH subgroups, PMMoV, and crAssphage was evaluated by principal component analysis (PCA) along with other microbial indicators, such as 16S ribosomal RNA gene markers of the *Bacteroidales* species. The viabilities (infectivity indices) of FRNA phage genogroups were estimated by comparing their numbers determined by infectivity-based and molecular assays. The PCA explained 58.2% of the total information and classified microbes into three groups: those considered to be associated with pig and human fecal contamination and others. Infective and gene of genogroup IV (GIV)-FRNA phage were assumed to be specific to pig fecal contamination, while the genes of GII-FRNA phage and crAssphage were identified to be specific to human fecal contamination. However, PMMoV, infective GI-FRNA phage, and FDNA phage were suggested to not be specific to human or pig fecal contamination. FRNA phage genogroups, especially the GIV-FRNA phage, were highly inactivated in the warm months in Japan (i.e., July to November). Comparing the infectivity index of several FRNA phage genogroups or other viruses may provide further insight into viral inactivation in the natural environment and by water treatments.

## Introduction

Enteric pathogens are contained in human and animal feces. The pathogens excreted from these animals are subjected to wastewater treatment. However, it is difficult to completely remove and inactivate all pathogens by the treatment, and the surviving pathogens are released into the water environment. Enteric viruses are known to be more resistant to water treatments than conventional fecal indicator bacteria, such as coliform bacteria and *Escherichia coli* (*E*. *coli*) [1–5]. Recreational activity in water environments such as rivers and lakes, where affected by untreated and treated wastewater, can be one of the causes of infection with enteric pathogens [6–8]. Some viruses, such as the pepper mild mottle virus (PMMoV), crAssphage, and F-specific bacteriophages (FPH), are expected to serve as indicators of viral contamination to complement the role of conventional fecal indicator bacteria [9–12].

Since the conventional fecal indicator bacteria cannot indicate fecal pollution sources, the microbial source tracking (MST) approach is used to identify sources of fecal pollution in water. Recently, 16S ribosomal RNA gene markers of host-specific *Bacteroidales*, which are predominantly present in human and animal gut flora, have been widely applied as MST tools [13–16]. PMMoV, crAssphage, and FPH subgroups, which are supposed to be specifically present in the feces of either or both humans and animals, are potentially useful MST tools [17–20].

PMMoV, a plant pathogen, is known to be highly abundant in human feces [20] and is considered to be a good indicator of human fecal contamination [17,21–23]. PMMoV is abundant in wastewater, surface water, and drinking water sources where human fecal pollution is present [22,24,25]. However, PMMoV was also detected in chicken, seagull [17], and pig feces samples [25].

CrAssphage is a bacteriophage that infects the human gut symbiont *Bacteroides intestinalis* [26]. Like PMMoV, crAssphage is abundant in the human gut and is regarded as a potential indicator of human fecal contamination [18]. CrAssphage has been identified in wastewater and surface water [27,28]. Previous studies have reported that crAssphage was detected in slurry from the abattoirs of pigs, cattle, and poultry [27]. Since PMMoV and crAssphage can be found in non-human feces, it is necessary to further investigate the host specificity of PMMoV and crAssphage.

FPH are abundantly present in human and animal feces. They are classified into FDNA phages (FDNAPHs) and FRNA phages (FRNAPHs). The occurrence and fate of FDNAPHs have not been studied as well as FRNAPHs. Previous studies have identified the occurrences of FDNAPHs in municipal wastewater and pig, cow, and gull feces [19,29]. FRNAPH is further classified into four genogroups, from genogroup I (GI) to GIV, which share size and morphology with enteric RNA viruses [30]. GI- and GIV-FRNAPHs are reported to be present in pig, cattle, and poultry feces [31]. GII- and GIII-FRNAPHs are present in human feces [19]. However, some studies have presented data that are not in agreement with these findings [31,32]. Consequently, the host specificity of FPH subgroups is still unclear. For the quantification of FRNAPH, reverse transcription (RT)-qPCR assays for each genogroup have been established [33]. Genes from both inactive and infective viruses are quantified by these assays. A quantitative assay for infective FRNAPH genogroups has also been developed [34]. In this assay, infective FRNAPH genogroups are propagated in liquid medium and detected via RT-PCR. By comparing these two assays, the viability of each FRANPH subgroup in the water environment can be estimated [35]. Therefore, FRNAPH subgroups can be one of the potential indicators for assessing the infectivity of viruses in the environment, which would be helpful for risk management [36].

The objective of this study is to evaluate the applicability of FPH subgroups, PMMoV, and crAssphage as indicators of fecal contamination and viral inactivation in river water affected by either or both human and pig feces. The host specificity of FRNAPH genogroups, FDNAPH, PMMoV, and crAssphage in the samples was evaluated by principal component analysis (PCA) along with other microbial indicators, such as 16S ribosomal RNA gene markers of host-specific *Bacteroidales*. Viabilities (infectivity indices) of FRNAPH genogroups were estimated by comparing their concentrations measured by the cultural and molecular assay.

## Materials and methods

### Sample collection

**Fig 1:**
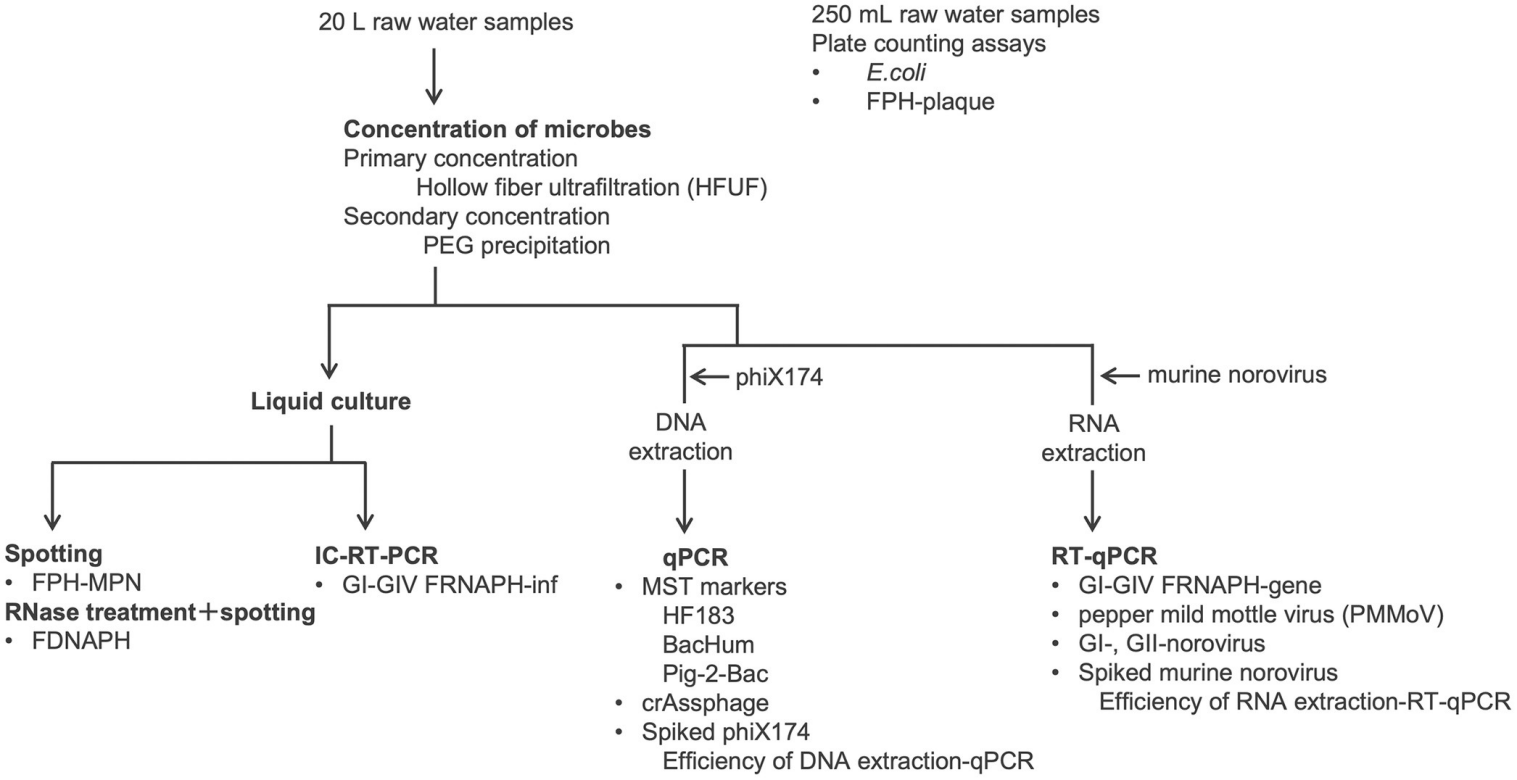
Flow diagram of sample processing for quantification of *E*. *coli*, FPH-plaque, FPH-MPN, FDNAPH, FRNAPH genogroups-inf (GI-GIV), MST markers; HF183, BacHum, Pig-2-Bac, crAssphage, FRNAPH-genogroups-gene (GI-GIV), pepper mild mottle virus (PMMoV), and noroviruses (GI and GII) in river water samples and spiked phiX 174 and murine norovirus by cultural and (RT-) qPCR assays. The entire process took place at a lab. in Toyama, Japan.

Surface water samples were collected monthly from November 2019 to November 2021 at three sites at the Oyabe River and its tributaries (O1, O2, and O3) and one site each at the Jinzu River and Sho River (J and S, respectively) in Toyama Prefecture, Japan (S2 Table). Access to the sampling sites and sample collection were not restricted, and no permits were required for the field work in this study. In April and May 2020, the samples at the five sites were not collected, and in June 2020, the sample at S was not collected due to the COVID-19 pandemic. Consequently, 108 surface water samples were collected. O1 is located in the mainstream of the Oyabe River. There are approximately 40,000 people around and upstream of O1. Most of them are served with a municipal wastewater treatment plant discharging its treated effluent downstream of O1. The rest of them are served with individual and combined septic tank systems, which discharge their treated effluent into the river and can contribute to human fecal pollution at O1. O2 is located at the subsidiary stream of the Oyabe River and approximately 3 km upstream of O1. O2 is affected by wastewater from a pig farm located approximately 3 km upstream. There are no households around and upstream of O2, and therefore, it is unlikely that sources other than effluent from septic tanks used by pig farm workers contribute to human fecal contamination at O2. O3 is located at the subsidiary stream of the Oyabe river that confluences downstream of O1. There is another pig farm upstream of O3. Sites J and S are affected by treated effluent from municipal wastewater treatment plants upstream. At each site, 20 L and 250 mL grab samples were collected in presterilized polyethylene container and bottle, respectively. The sample in the polyethylene container (20 L) was subjected to a concentration and quantification of microbes, while that in the polyethylene bottle (250 mL) was subjected to plate counting assays for *E*. *coli* and FPH within 24 h after the collection(Fig 1).

### Concentration of microbes

The sample in the 20 L polyethylene container was concentrated by hollow fiber ultrafiltration (HFUF) cartridge (APS-25SA; Asahi Kasei Medical Co., Osaka, Japan) and following centrifugation and polyethylene glycol (PEG) precipitation, as previously described [37–39]. Prior to the sample filtration, the HFUF was blocked by circulating 200 mL of fetal bovine serum (FBS) in the cartridge for 2 minutes and incubated overnight. After removing the remaining FBS, 20 L of the sample was passed through the HFUF cartridge. After filtration, the retentate (about 100–200 mL) was back-flushed into a collection bottle and mixed with a 100 × elution buffer (10% Tween 80, 1% sodium polyphosphate, 0.1% antifoam A). The mixture was then circulated in the HFUF cartridge for 2 minutes to further recover the bacteria and viruses remaining in the cartridges; then, it was finally recovered as a primary concentrate. The primary HFUF concentrate was centrifuged at 3,000 × g for 5 minutes, and the supernatant was subjected to a secondary concentration by PEG precipitation [39]. The supernatant was mixed with PEG8000 and sodium chloride at final concentrations of 10% and 5.8% (w/v), respectively. The mixture was incubated overnight on a shaker at 4°C. Subsequently, the mixture was centrifuged at 10,000 × g for 30 minutes. After discarding the supernatant, the pellet was resuspended by 500 μL of phosphate buffer per 40 mL of the supernatant and collected as a secondary concentrate. The secondary concentrate was immediately subjected to the liquid culturing for integrated culture RT-PCR (IC-RT-PCR) and then frozen at -80°C. The frozen concentrate was further subjected to RNA and DNA extraction processes within 6 months.

### RNA or DNA extraction

Murine norovirus (MNV) and coliphage phiX 174 were used as molecular process controls (MPCs) to evaluate potential underestimations of gene concentrations due to impurities during RNA extraction-RT-qPCR and DNA extraction-qPCR processes, respectively.

Briefly, 140 μL of the secondary concentrate was spiked with 2 μL of MNV (8.5 × 10^5^ copies/μL) and subjected to RNA extraction using a QIAamp viral RNA mini kit (Qiagen) to obtain a 60 μL of RNA extract, in accordance with the manufacturer’s instructions.

Regarding DNA extraction, the secondary concentrate and pellet obtained by centrifuging the primary concentrate were mixed in a ratio that reproduced the ratio in the original sample. For example, the resultant volumes of the secondary concentrate and the pellet were 2.0 mL and 1.0 mL, respectively; they were mixed in a ratio of 2:1. Then, 100 μL of the mixture was spiked with 5 μL of phiX 174 (8.0 × 10^4^ copies /μL) and subjected to DNA extraction using ISOSPIN Blood & Plasma DNA (NIPPON GENE) to obtain 100 μL of DNA extract, in accordance with the manufacturer’s instructions.

The RNA and DNA extracts were diluted 10-fold with nuclease-free water to mitigate the effect of (RT-) PCR inhibition. Both the 10-fold diluted and undiluted RNA and DNA extracts were subjected to (RT-) qPCR assays. The spiked MNV, indigenous FRNAPH genogroups (GI-GIV-FRNAPH-gene), PMMoV, GI-Norovirus (NoV), and GII-NoV were quantified by RT-qPCR assays. The spiked phiX 174, host-specific *Bacteroidales* 16S ribosomal RNA gene markers (HF183, Pig-2-Bac, and BacHum), and crAssphage were quantified by qPCR assays. MPCs were also spiked into nuclease free water and subjected to nucleic acid extraction processes as the samples. These extracts were used for positive control and no template control, which did not show any positive signal, for MPCs and other target genes, respectively. DNA and RNA extracts were stored at 4°C for up to 1 month.

### (RT-) qPCR assay

(RT-) qPCR assays were performed with a qTOWER^3^ (analytik Jena). The sequences of primers and TaqMan^®^ probes were derived from previous studies (S1 Table) [15,33,40–46].

To quantify viral RNA, one-step RT-qPCR was performed using a One Step PrimeScript^™^ RT-PCR Kit (Perfect Real Time) (TaKaRa). A reaction mixture (25 μL) was prepared by mixing 2.5 μL of the RNA extract, 12.5 μL of a 2 × One Step RT-PCR Buffer III, 0.5 μL of a TaKaRa Ex Taq HS, 0.5 μL of a PrimeScript RT enzyme Mix II, 400 nM each of forward and reverse primers, 150 nM of a TaqMan^®^ probe, and nuclease-free water. The reaction was performed under the following thermal cycling conditions: RT reaction at 42°C for 5 minutes, initial denaturation and inactivation of the RT enzyme at 95°C for 10 seconds, followed by 50 cycles of amplification with denaturation at 95°C for 5 seconds, and annealing and extension at specific temperatures for each assay (S1 Table) for 30 seconds.

For the quantification of microbial DNA, qPCR was performed. A reaction mixture (25 μL) was prepared by mixing 2.5 μL of the DNA extract, 12.5 μL of a TaqMan^™^ Gene Expression Master Mix (Thermo Fisher Scientific), 400 nM each of forward and reverse primers, 150 nM of a TaqMan probe, and nuclease-free water. qPCR was performed under the following thermal cycling conditions: DNA polymerase activation at 50°C for 2 minutes and 95°C for 10 minutes, followed by 50 cycles of amplification with denaturation at 95°C for 10 seconds and annealing and extension at specific temperatures for each assay(S1 Table) for 30 seconds.

To obtain a calibration curve, a 10-fold serial dilution (concentrations ranged from 1.0 × 10^0^ to 1.0 × 10^5^ copies/reaction) of standard RNA or standard DNA containing the target sequence were subjected to (RT-) qPCR. If the resultant cycle threshold (Ct) value from a sample was corresponding to > 1 copy/reaction, the sample was determined to be positive for the target microbes. Absence of the positive signal in the no template control was confirmed in every (RT-) qPCR run to identify the potential contamination of the template into the reagents.

### Quantification of Infectious FRNAPH Genogroups and FDNAPHs

IC-RT-PCR coupled with the most probable number (MPN) approach [34] was applied to quantify infectious FRNAPH genogroups (GI-, GII-, GIII-, and GIV-FRNAPH-inf). For the IC-RT-PCR, 40, 4, 0.4, and 0.04 μL of the secondary concentrate were subjected to FPH propagation in triplicate for each volume. Each volume of the sample was mixed with 40 μL of tryptone glucose broth (TGB) containing *Salmonella typhimurium* WG49 (WG49) at the exponential growth phase, 20 mg/L of kanamycin, and 100 mg/L of nalidixic acid and was incubated overnight at 37°C. Presence/absence of infectious FRNAPH genogroups in each volume of the concentrate were determined by RT-PCR. To extract RNA, 3 μL of the sample culture was incubated at 95°C for 5 minutes. The RNA extract was then subjected to one-step RT-PCR using the One Step PrimeScript^™^ RT-PCR Kit (Perfect Real Time) (TaKaRa), as described above. An infectious FRNAPH genogroup was considered positive if the resultant Ct value was at least 3.3 lower than that would be obtained with the conventional RT-qPCR, i.e., if the number of phages increased > 10-fold by the liquid culture process, and was less than 30. Otherwise, the amplification curve could have been attributed to inactive FRNAPHs, which could not multiply during the liquid cultivation process. The concentration of infectious FRNAPH genogroups was determined by referring to an MPN table for three 10-fold dilutions with three tubes at each dilution, provided by Blodgett (2010) [47].

For detection of FPH and FDNAPH by a spotting assay, tryptone glucose agar (TGA) prepared by adding agar powder into TGB was distributed onto the plates and solidified. Then, 3 μL each of the sample cultures was dropped on it and incubated overnight at 37°C. The concentration of FPH (FPH-MPN) in the sample was determined by the MPN approach based on presence/absence of FPH in each sample culture determined based on plaques formed at the spotted area. Quantification of FDNAPH was conducted in the same manner, using TGA containing 200 mg/L of RNase (Sigma Aldrich).

### Quantification of Active *E*. *coli* and FPH by plate-counting assays

Raw 50 mL samples were subjected to the plate counting assays to determine the number of colony forming units (CFU) of *E*. *coli*, and the plaque-forming unit (PFU) of FPH. *E*. *coli* in the sample was quantified with Chromocult^®^ Coliform Agar (Merck Darmstadt, Germany). In addition to the liquid cultivation-based assays described in the previous section, FPH was quantified by the conventional plaque assay (FPH-plaque), according to a previous study that employed *S*. *Typhimurium* WG49 as the host strain [48].

### Determining the efficiencies of nucleic acid extraction-(RT-) qPCR

In this study, MNV and phiX 174 were used as molecular process controls, and their detection efficiencies were determined to estimate efficiencies of the RNA extraction-RT-qPCR and DNA extraction-qPCR processes, respectively. The detection efficiencies were estimated by comparing the observed gene concentration of MPC in nuclease-free water with that in the samples; namely, the detection efficiency (R) was calculated as follows:

$$\text{R}={\text{C/C}}_{0}\times 100$$

where C represents the observed gene concentration of MPC in a sample, and C_0_ represents the observed gene concentration of MPC in the nuclease-free water.

### Statistical analysis

Principal component analysis (PCA) and cluster analysis were conducted using IBM SPSS Statistics version 22 and R (version 4.2.0) respectively to evaluate host-specificity of the microbes among the collected samples. PCA was performed using a correlation matrix with a varimax rotation, retaining principal components (PCs) whose eigenvalues were greater than 1 [49]. To reduce the potential negative effects of non-detected data, the microbes that showed positive rates of 24% or higher out of all the analyzed samples were selected for analysis (i.e., HF183, Pig-2-Bac, GI-, GII-, and GIV-FRNAPH-inf, GII- and GIV-FRNAPH-gene, crAssphage, PMMoV, *E*. *coli*, FPH-plaque, FPH-MPN, and FDNAPH). The non-detected results were replaced with values half the detection limits of the (RT-) qPCR, IC-RT-PCR, colony, and plaque assays (the detection limit values were approximately 1.0 × 10^0^ copies/100 mL, 1.0 × 10^−1.2^ MPN/100 mL, 1.0 × 10^0.9^ CFU/100 mL, and 1.0 × 10^0.3^ PFU/100 mL, respectively), respectively. Prior to the analysis, the log_10_-transformed data for each microbial target were normalized by subtracting and dividing their mean and standard deviation values, respectively. The normalized data was also used for the cluster analysis.

Multiple comparison tests using Tukey’s honest significant differences test (HSD) and the Games-Howell method were conducted to compare the geometric mean concentrations of microbes at different sites. The geometric mean concentrations were calculated after excluding non-detected data. The multiple comparison test conducted only the data at sites where the microbe was detected at least thrice (i.e., HF183, BacHum, Pig-2-Bac, GI- and GII-FRNAPH-inf, GII-FRNAPH-gene, crAssphage, PMMoV, *E*. *coli*, FPH-plaque, FPH-MPN, and FDNAPH). Prior to the multiple comparison tests, an unpaired one-way ANOVA was conducted. If the one-way ANOVA indicated that the concentrations of each microbe are homoscedastic (*p* < 0.05), Tukey’s HSD method was used as a post-hoc comparison; otherwise, the Games-Howell method was used.

A chi-square test was conducted to compare the positive rate of microbes at different sites. The significance value was corrected to *p* < 0.005 to account for the number of comparisons by the Bonferroni method.

## Results

### Detection efficiency of MPCs

To determine the efficiency of RNA extraction-RT-qPCR, MNV was spiked into the samples as a control and recovered by RT-qPCR from undiluted and 10-fold diluted RNA extracts. The detection efficiency of the spiked MNV was < 10% in 24 of the 108 undiluted RNA extracts. The detection efficiency in 8 out of the 24 extracts was improved to > 10% by 10-fold diluting the RNA extract, while the efficiency of the remaining 16 extracts was not noticeably improved by the dilution. These 16 samples are supposed to be affected by inefficient RNA extraction rather than RT-qPCR inhibition. Similarly, the detection efficiency of phiX 174, which was spiked to estimate the efficiency of DNA extraction-qPCR, resulted in < 10% in 14 out of the 108 undiluted DNA extracts. The detection efficiencies in 4 out of the 14 extracts were improved to > 10% by 10-fold diluting the DNA extract, while those in the remaining 10 samples were not noticeably improved by the dilution. These 10 samples are supposed to be affected by inefficient DNA extraction rather than qPCR inhibition. Regardless of the detection efficiencies, both undiluted and 10-fold diluted RNA and DNA extracts were subjected to microbial gene quantification by (RT-) qPCR, and one showed a higher observed concentration was selected. In this study, phiX174 was selected as the MPC because it is easy to handle and widely used as a model for enteric viruses [30]. A possible limitation of phiX 174 as the MPC is that it is an ssDNA virus, while dsDNAs in crAssphage and bacteria were targeted in this study. The representativeness of phiX 174 to these microbes in the DNA extraction process needs to be clarified in the future. It is known that the sensitivity to (RT-) PCR inhibitor depends on PCR assays [50]. Therefore, even if the MPCs could be efficiently quantified, quantification of other microbes can be underestimated. Considering this, in this study, both the 10-fold diluted and undiluted extracts were subjected to (RT-) qPCR assays for all the targets, and the one with a higher observed concentration was selected.

### Occurrence of MST markers

**Fig 2:**
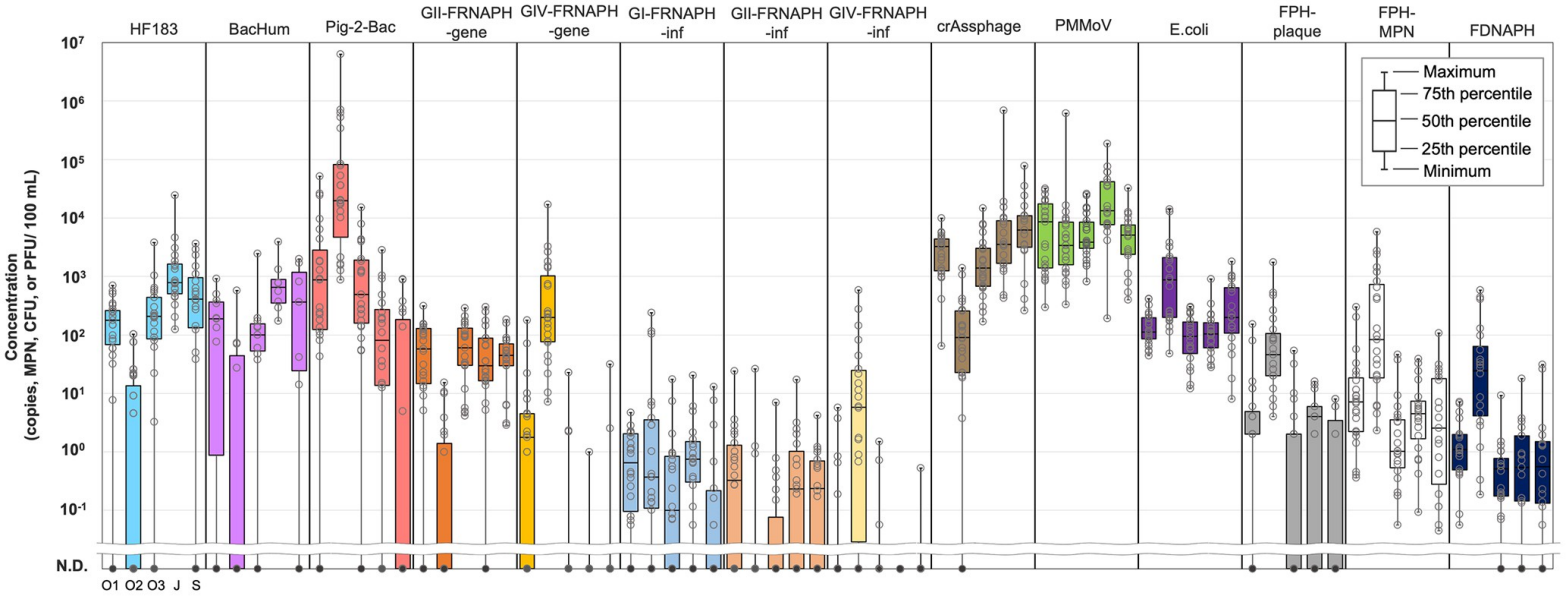
Concentrations of microbes in river water samples. The concentrations of microbes that showed a 24% or higher positive rate (i.e., HF183, BacHum, Pig-2-Bac, GI-, GII- and GIV- FRNAPH-inf, GII- and GIV-FRNAPH-gene, crAssphage, PMMoV, *E*. *coli*, FPH-plaque, FPH-MPN, FDNAPH). HF183, Pig-2-Bac and crAssphage were detected by qPCR (copies/100 mL). GII- and GIV-FRNAPH-gene and PMMoV were detected by RT-qPCR (copies/100 mL). GI-FRNAPH-inf, GII- and GIV-FRNAPH-inf were detected MPN-IC-RT-PCR (MPN/100 mL). *E*. *coli* and FPH-plaque were detected by plate counting assays (CFU/100 mL and PFU/100 mL, respectively). FPH-MPN and FDNAPH were detected by spotting (MPN/100 mL). “N.D.” on the vertical axis indicates “not detected”. The box and whisker plots indicate the maximum, minimum, 75th, 50th, and 25th percentile values. The total number of samples is 108 but 60, 5, and 14 samples were not used for quantifications of BacHum, GI-GIV-FRNAPH-inf, and FDNAPH, respectively.

Table 1 and Fig 2 summarize the positive rates and observed concentrations, respectively, of each microbial target. The observed concentrations were calculated considering concentration factors and tested volumes but the detection efficiency of MPCs. GI-GIV-FRNAPH-inf in 5 samples collected in June 2021 was not quantified due to a problem with host growth. Similarly, FDNAPH in 14 samples collected in February, March and June 2020 was not quantified because the samples were used up before the analysis. BacHum in the samples collected after December 2020 was not quantified because HF183 showed higher detected concentration and detection frequency, indicating that BacHum is less informative, as described below. HF183, BacHum, and Pig-2-Bac were detected in 81% (88/108), 75% (36/48), and 82% (89/108) of the samples, respectively. At all sites except for O2, HF183 was detected with positive rates of 89–100%. The geometric mean concentration of HF183 at J was significantly higher than that at O1, O2, and O3 (Games-Howell, *p* < 0.05). On the other hand, at O2, HF183 was detected in 35% (8/23) and showed a significantly lower observed concentration than the other four sites. BacHum tended to be detected in slightly lower concentrations than HF183 at each site and showed a lower positive rate. Pig-2-Bac was detected in all samples (23/23) collected at O2 with a geometric mean concentration (10^4.4^ copies/ 100 mL) significantly higher than at the other four sites (Games-Howell, *p* < 0.05). At O1 and O3, Pig-2-Bac was detected at relatively high frequencies—91% (21/23) and 96% (22/23), respectively. At J and S, Pig-2-Bac was detected in relatively low frequencies—80% (16/20) and 37% (7/19), respectively, with geometric mean concentrations (10^2.1^ copies/100 mL and 10^2.3^, respectively) significantly lower than that at O2 (Games-Howell, *p* < 0.05).

**Table 1:** Positive rates of microbial targets in the samples.

positive rate (%)	O1		O2		O3		J		S		Total	
HF183	96%	22/23	35%	8/23	91%	21/23	100%	20/20	89%	17/19	81%	88/108
BacHum	73%	8/11	36%	4/11	91%	10/11	100%	8/8	86%	6/7	75%	36/48
Pig-2-Bac	91%	21/23	100%	23/23	96%	22/23	80%	16/20	37%	7/19	82%	89/108
GI-FRNAPH-gene^a^	0%	0/23	0%	0/23	0%	0/23	0%	0/20	0%	0/19	0%	0/108
GII-FRNAPH-gene	91%	21/23	30%	7/23	100%	23/23	95%	19/20	100%	19/19	82%	89/108
GIII-FRNAPH-gene	9%	2/23	4%	1/23	0%	0/23	10%	2/20	0%	0/19	5%	5/108
GIV-FRNAPH-gene	61%	14/23	100%	23/23	13%	3/23	5%	1/20	11%	2/19	40%	43/108
GI-FRNAPH-inf^b^	86%	19/22	77%	17/22	64%	14/22	89%	17/19	44%	8/18	73%	75/103
GII-FRNAPH-inf	59%	13/22	14%	3/22	27%	6/22	58%	11/19	61%	11/18	43%	44/103
GIII-FRNAPH-inf	0%	0/22	5%	1/22	5%	1/22	0%	0/19	0%	0/18	2%	2/103
GIV-FRNAPH-inf	23%	5/22	73%	16/22	14%	3/22	0%	0/19	6%	1/18	24%	25/103
crAssphage	100%	23/23	96%	22/23	100%	23/23	100%	20/20	100%	19/19	99%	107/108
PMMoV	100%	23/23	100%	23/23	100%	23/23	100%	20/20	100%	19/19	100%	108/108
GI-NoV	0%	0/23	0%	0/23	0%	0/23	0%	0/20	0%	0/19	0%	0/108
GII-NoV	0%	0/23	0%	0/23	0%	0/23	0%	0/20	0%	0/19	0%	0/108
*E*. *coli*	100%	23/23	100%	23/23	100%	23/23	100%	20/20	100%	19/19	100%	108/108
FPH-plaque	83%	19/23	100%	23/23	39%	9/23	70%	14/20	42%	8/19	68%	73/108
FPH-MPN	100%	23/23	100%	23/23	100%	23/23	100%	20/20	100%	19/19	100%	108/108
FDNAPH	100%	20/20	100%	20/20	95%	19/20	82%	14/17	82%	14/17	93%	87/94

^*a*^: Gene quantification assay

^*b*^: Infectivity assay.

### Occurrences of FRNAPH-gene and FRNAPH-inf

GI-FRNAPH-gene was not detected at any of the sites. GII-FRNAPH-gene was detected in 82% (89/108) of the samples. GIII-FRNAPH-gene was detected at a relatively low frequency (5% (5/108)), while GIV-FRNAPH-gene was detected in 40% (43/108) of the samples. At O2, GII-FRNAPH-gene was detected in a frequency of 30% (7/23), while it was detected in 91–100% of the samples collected at other sites. Moreover, the geometric mean concentration of GII-FRNAPH-gene at O2 (10^0.63^ copies/100 mL) was significantly lower than that at other sites (*p* < 0.05, Games Howell). GIII-FRNAPH-gene was detected only in samples at O1 (9% (2/23)), O2 (4% (1/23)), and J (10% (2/20)). At O2, GIV-FRNAPH-gene was detected in all the samples (23/23) with a significantly higher geometric mean concentration (10^2.4^ copies/100 mL) than at other sites (*p* < 0.05, Games Howell). GIV-FRNAPH-gene was also detected in a relatively high frequency of 61% (14/23) at O1, while it was detected in lower frequencies of 5–13% at O3, J, and S.

GI-FRNAPH-inf was detected in 73% (75/103) of all samples, with detection rates at each site other than S ranging from 64% to 89%. At S, it was detected in a significantly lower frequency (44% (8/18)) than at O1 and J (*p* < 0.005, chi-square test). GII-FRNAPH-inf was detected in 43% (44/103) of all samples, with detection rates at each site other than O2 ranging from 27% to 61%. At O2 it was detected in a significantly lower frequency (14%, 3/22) than at the other sites (*p* < 0.005, chi-square test). For GI- and GII-FRNAPH-inf, no significant differences in geometric mean concentrations among the sites were observed (*p* < 0.05, Tukey). At O3, GII-FRNAPH-inf was detected in 27% of the samples (6/22), while GII-FRNAPH-gene was detected from all the samples. GIII-FRNAPH-inf was detected in only 2% (2/103) of all samples. GIV-FRNAPH-inf was detected in 24% (25/103) of all samples and in 0–23% of the samples at sites other than O2, where it was detected at a significantly higher frequency (73%, 16/23) than at other sites (*p* < 0.005, chi-square test).

### Occurrences of CrAssphage, PMMoV, and NoVs

CrAssphage was detected in 99% (107/108) of the samples. PMMoV was detected in all the samples (n = 108). GI- and GII-NoVs were not detected in any of the samples. The geometric mean concentrations of crAssphage did not differ significantly among the sites (10^3.2^–10^3.7^copies/100 mL) except for O2 (10^2.0^ copies/100 mL) (Games-Howell, *p* < 0.05). The geometric mean concentration of PMMoV at J (10^4.2^ copies/100 mL) was the highest among the sites and was significantly higher than that at sites O2, O3, and S (Games-Howell, *p* < 0.05). There was no significant difference in PMMoV geometric mean concentration in other pairs of sites (Games-Howell, *p* < 0.05).

### Occurrences of *E*. *coli*, FPH, and FDNAPH

*E*. *coli* was detected in 100% (108/108) of the samples. FPH-MPN and FPH-plaque were detected in 100% (108/108) and 68% (73/108) of the samples, respectively. FDNAPH was detected in 93% (87/94) of the samples tested. The geometric mean concentrations of *E*. *coli*, FPH-MPN, FPH-plaque, and FDNAPH at O2 were significantly higher than those at other sites (*p* < 0.05, *E*. *coli*, FPH-MPN, and FDNAPH: Tukey HSD, FPH-plaque: Games-Howell).

### Microbial Characterization by PCA

**Fig 3:**
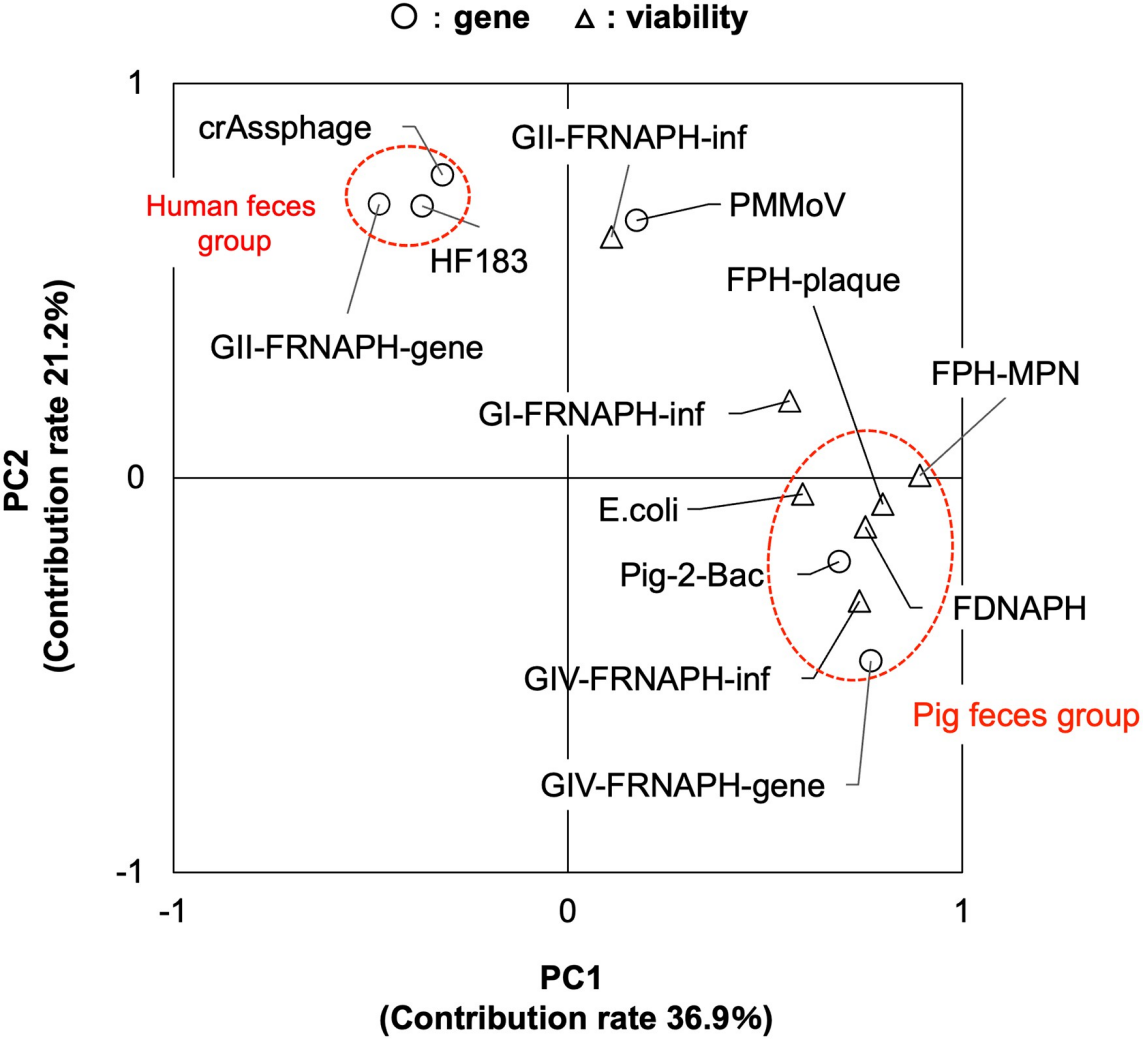
Principal component analysis (PCA) of observed concentrations of each microbial target in the samples (n = 94). The analysis employs concentrations of microbial targets with 24% or higher positive rates (i.e., HF183, Pig-2-Bac, GI-, GII- and GIV-FRNAPH-inf, GII- and GIV-FRNAPH-gene, crAssphage, PMMoV, *E*. *coli*, FPH-plaque, FPH-MPN, and FDNAPH). Circles (○) and triangles (Δ) refer to the indicators quantified based on gene and viability, respectively. The vertical and horizontal axes indicate principal components (PC) 1 and 2, which explained 36.9% and 21.2% of the total information, respectively.

To study the host specificity of potential indicators, PCA was employed. By this PCA, 58.1% of the total information in the data could be explained (PC1: 36.9%, PC2: 21.2%) (Fig 3). The microbial targets were roughly separated into three groups based on the distribution of the plots in Fig 3. In the fourth quadrant, especially in an area of x > 0.6 and y < 0.0, GIV-FRNAPH-gene and -inf, *E*. *coli*, FPH-plaque, FPH-MPN, and FDNAPH were plotted with Pig-2-Bac, suggesting their close relationship with porcine contamination. Among them, FDNAPH was most closely plotted to Pig-2-Bac. In the second quadrant, especially at around x = -0.4/ y = 0.7, GII-FRNAPH-gene and crAssphage were plotted with HF183, suggesting a close relationship with human contamination. In the first quadrant, other microbes, namely, GI-FRNAPH-inf, GII-FRNAPH-inf, and PMMoV, were plotted. The PCA in our study explained only 58.1% (PC1: 36.9%, PC2: 21.2%) of the total information. This might be caused by the third group of microbes (GI- and GII-FRNAPH-inf and PMMoV), which were not classified into either the pig group or the human group. By excluding the third-group data, the percentage of information explained by PCA was improved to be 67.5% (PC1: 40.0%, PC2: 27.5%) (S2 Fig).

### FDNAPH/FPH-MPN concentration ratios

**Fig 4:**
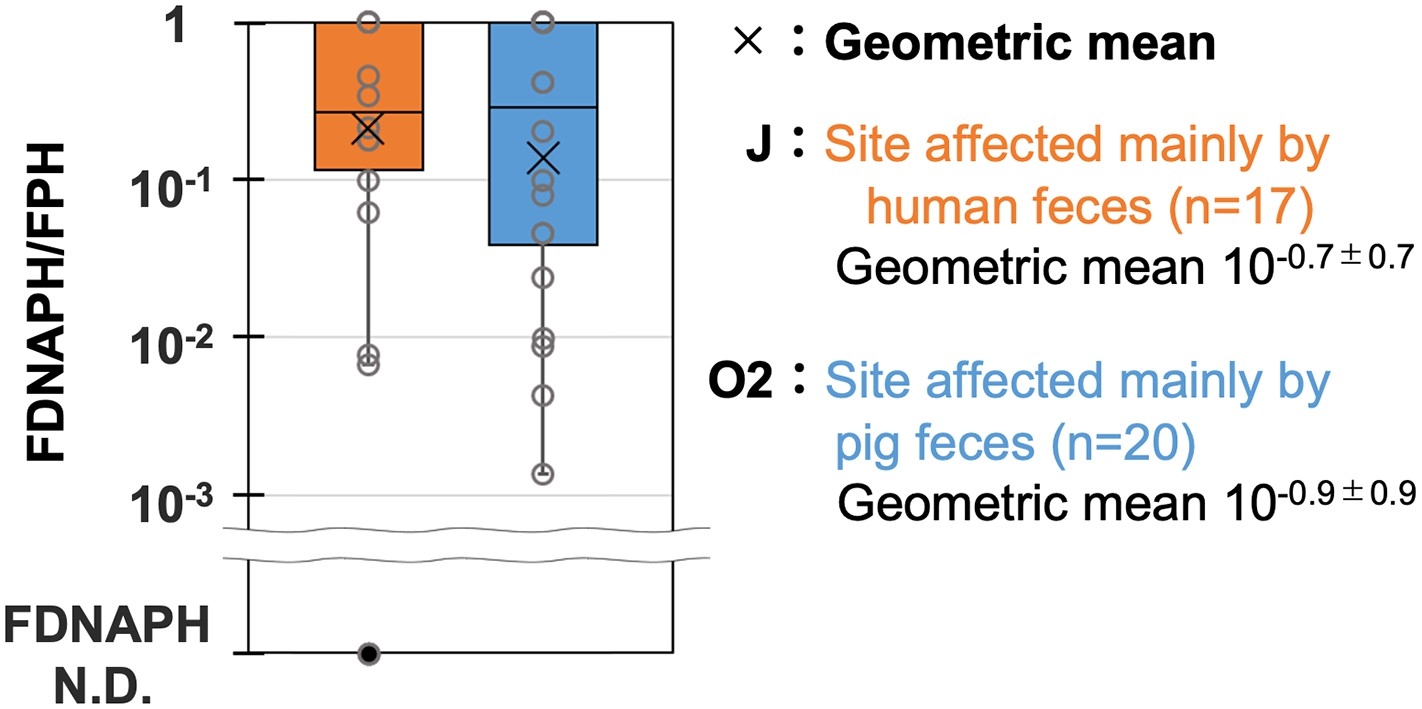
FDNAPH/FPH-MPN concentration ratios (FDNAPH/FPH-MPN) at J and O2. J (n = 17) and O2 (n = 20) are supposed to be affected mainly by human feces and by pig feces, respectively. Circles represent each sample. The cross mark (×) indicates the geometric mean value.

To clarify the host specificity of FDNAPH, which was suggested to be more associated with pig feces by PCA, FDNAPH/FPH-MPN concentration ratios (FDNAPH/FPH-MPN) were compared in Fig 4. The geometric means of FDNAPH/FPH at J (10^−0.7 ± 0.7^) and O2 (10^−0.9 ± 0.9^), which are supposed to be affected mainly by human and pig feces, respectively, were almost equivalent.

### Infectivity indices of FRNAPH genogroups

**Fig 5:**
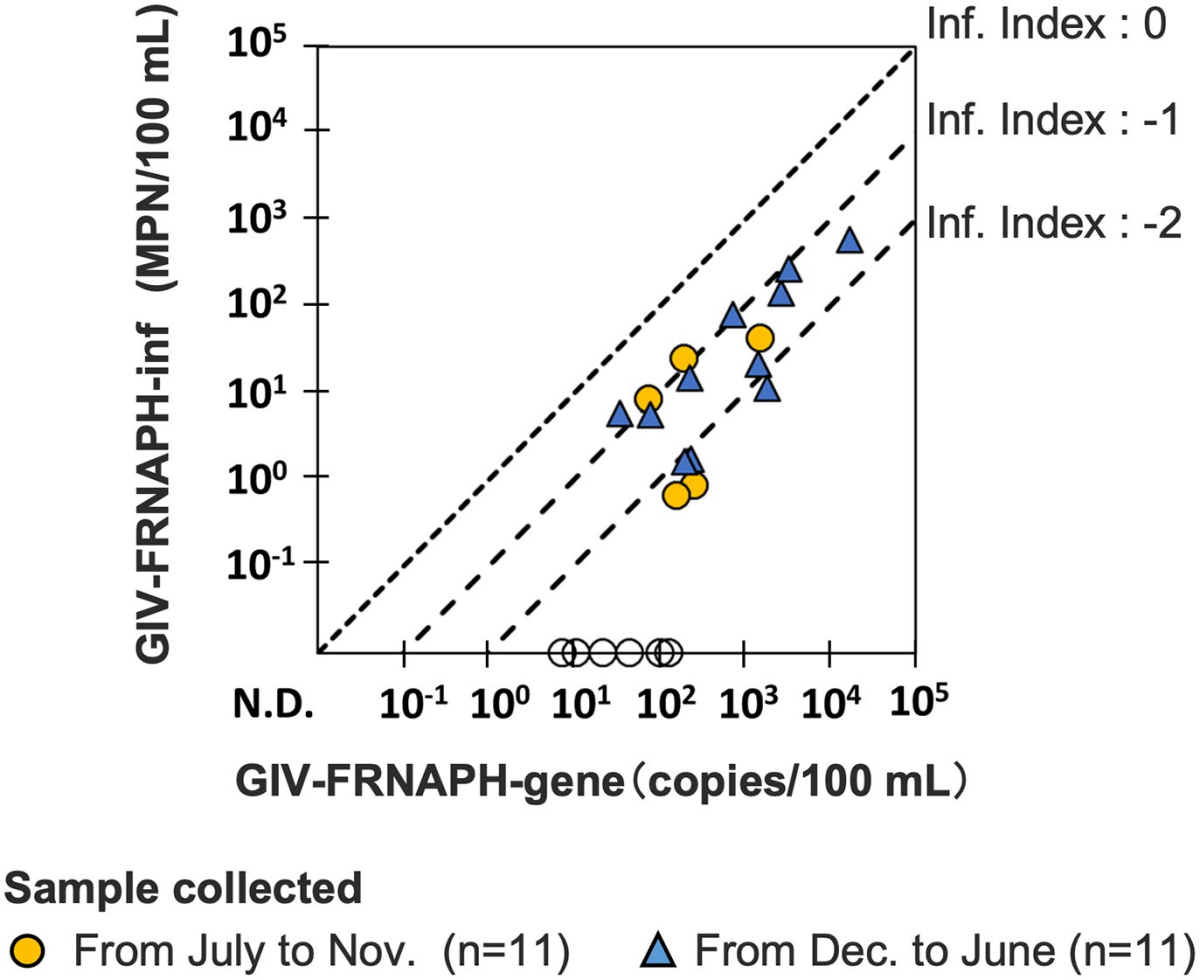
The infectivity index (Inf. index) of GIV-FRNAPHs in the surface water samples at O2 (n = 22). The infectivity index was defined as the differences between the log_10_-transformed concentrations of infectious GIV-FRNAPHs (MPN) and GIV-FRNAPH-gene (copies) as indicated by diagonal lines. Circles (○) and triangles (Δ) represent samples collected during the warm months (from July to November) and during the cool month (from December to June), respectively. “N.D.” on the axis means “not detected” and white plots on the axis indicate that the sample was negative in cultural and/or RT-qPCR assays.

The infectivity and gene concentrations of the FRNAPH genogroups in the samples were determined via MPN-IC-RT-PCR and RT-qPCR assays, respectively. The observed concentrations of infectivity and gene of GII-FRNAPH at sites other than O2 and GIV-FRNAPH at O1 and O2, whose detection rates were 30% or higher, were compared (Fig 5 and S1 Fig). In Fig 5, the infectivity index, which is defined as log_10_-transformed ratio of the concentration determined by infectivity assay (MPN-IC-RT-PCR) to the concentration determined by gene quantification assay (RT-qPCR), is also indicated. Regarding GIV-FRNAPH at O2, six samples were found to be positive only by RT-qPCR assay, suggesting that GIV-FRNAPH in the samples was highly inactivated. Notably, all six samples were collected in the warm months (July to November). At O1, GIV-FRNAPH tended to show higher infectivity index values during the cool months (December to June) (S1 Fig). The infectivity index of GII-FRNAPH showed variable values between -3.1 and 0.4 log_10_ regardless of the season and site (S1 Fig).

## Discussion

### Contamination sources at each site implied by MST markers

Previous studies on MST markers have reported high sensitivity (100%) of Pig-2-Bac to pig feces [15,51]. HF183 and BacHum have also been reported to exhibit high sensitivities (77–100%) to municipal wastewater samples [40,52,53] and human feces [54–56]. In this study, HF183 and BacHum exhibited similar trends at each site. The forward primer for HF183 shares 16 bases with that of BacHum, and these two markers target the same region on the 16S rRNA gene [40,41,53]. Considering that HF183 showed a higher detection rate and concentration, we mainly focused on HF183 as an MST marker of human fecal contamination in this study. At O2, Pig-2-Bac was detected from all the samples with a concentration significantly higher than those at the other four sites (10^4.6^ copies/100 mL). This strongly suggests that O2 is affected by wastewater from a pig farm upstream. HF183 was also detected at O2, but its positive rate and concentrations were low (35% (8/23) and < 2.0 log_10_ copies/100 mL, respectively). This implies that pig farm workers contributed to the contamination at O2, but the effect was limited. At O1, both HF183 and Pig-2-Bac were detected at high frequencies (96% and 91%, respectively) and concentrations. This implies that O1 was affected by both human and pig feces. Considering that the concentration of Pig-2-Bac at O1 was lower than that at O2, the porcine contamination at O1 was probably attributed to O2. Similarly, O3 and J, where Pig-2-Bac and HF183 were detected at high frequencies, seem to be affected by both municipal wastewater and pig farm wastewater. At S, HF183 was detected in a frequency of 89% (17/19) and concentration comparable to sites other than O2, while Pig-2-Bac was not frequently detected (37%, 7/19). This implies that S was mainly affected not by pigs but by municipal wastewater.

### Host specificity of FRNAPHs

In PCA, GIV-FRNAPH-gene and -inf were plotted near Pig-2-Bac, suggesting that GIV-FRNAPH was excreted specifically from pigs. Similarly, GII-FRNAPH-gene was plotted near HF183, suggesting that GII-FRNAPH was excreted specifically from humans. These highlight the efficacy of GIV-FRNAPH and GII-FRNAPH-genes as indicators of pig and human fecal contamination, respectively. In contrast, GII-FRNAPHs-inf was not classified in either the human feces group or the pig feces group. This is because GII-FRNAPHs-inf was detected in low frequencies and concentrations even at sites affected mainly by humans, especially at O3. Analysis of the infectivity index strongly suggested that the GII-FRNAPHs in some samples were highly inactivated. This resulted in low detection frequencies. In the PCA in this study, GI-FRNAPH-inf was classified into neither human nor pig feces groups. In general, GI-FRNAPH is regarded as an indicator of non-human fecal contamination [31]. However, GI-FRNAPH was reported to be a predominant FRNAPH genogroup in treated municipal wastewater, even though it was a minor genogroup before wastewater treatment [57,58]. These findings suggest that GI-FRNAPH cannot be a useful MST tool.

### Host specificity of CrAssphage and PMMoV

CrAssphage and PMMoV have been reported to be predominantly present in human feces [20,59]. PMMoV is derived from pepper products and is excreted in human feces. In the PCA in this study, crAssphage was plotted near HF183. This indicates that crAssphage is a potentially useful indicator of human fecal contamination, in accordance with a previous study [18]. On the other hand, PMMoV was classified into neither the human nor pig feces groups. This is probably because PMMoV was detected in all the samples, and its observed concentration at the site predominantly affected by pig (O2) was comparable to that at other sites, excluding J. A previous study reported that PMMoV was frequently detected in pig feces because pigs are usually fed leftover human food [51]. These findings suggest that PMMoV is not useful for a specific indicator of human fecal contamination.

### Host specificity of FDNAPH

*E*. *coli* and FPH are abundant in both human and other mammalian feces. *E*. *coli*, FPH-plaque, and FPH-MPN were plotted near Pig-2-Bac, which was predominantly detected at O2, in the PCA (Fig 3). This is mainly because they were detected at relatively low concentrations at sites suggested to be strongly affected by human feces (J and S). It is obvious that they are present in human feces in high concentrations. This result can be attributed to the difference in fecal strength at O2 and other sites. FDNAPH was also plotted near Pig-2-Bac. However, FDNAPH/FPH at J and O2 were almost equivalent, suggesting that the proportion of FDNAPH among FPH populations does not differ in human and pig feces. Therefore, like *E*. *coli* and FPH, FDNAPH was plotted near Pig-2-Bac probably due to the difference in fecal strength at O2 and other sites. Furthermore, FDNAPH cannot be regarded as an MST tool to discriminate between human and pig fecal pollution, although a previous study has shown that FDNAPH was significantly more abundant in municipal wastewater than pig feces [19].

### Infectivity indices of FRNAPH genogroups

The gene quantification assay (RT-qPCR) tends to show higher concentrations of FRNAPHs compared to the infectivity assay (IC-RT-PCR). This is because the gene quantification assay can detect both infectious and inactive viruses, while the infectivity assay can detect only infectious viruses. Thus, the degree of virus inactivation can be estimated by comparing the concentrations of genes and infectivity. GI-FRNAPH-gene was not detected in all samples, although GI-FRNAPH-Inf was detected in 73% of the samples. The sample volume subjected to RT-qPCR corresponds to approximately 50 ml of a raw water sample, while the volume subjected to IC-RT-PCR corresponds to approximately 2.6 L of a raw water sample. This difference in the test volume probably contributed to the result. The concentration of GI-FRNAPH-gene should be > 2 copies/100 mL to be detected, even if the detection efficiency was 100%. Considering that GI-FRNAPH-inf was < 10 MPN/100 mL in most of the samples, the result is understandable (Fig 2). GI-FRNAPH-Inf was the most frequently detected genogroup among the infectious FRNAPH genogroups. In addition, a previous study has shown that GI-FRNAPHs are more resistant to higher temperatures, high pH, and chlorination than other genogroups [60]. Therefore, GI-FRNAPH is the most stable genogroup in the natural environment. At O1 and O2, the infectivity index of GIV-FRNAPH tended to be lower during the warm months, although the trend was less clear at O1. In previous studies, infectious GIV-FRNAPH was detected only in winter in raw wastewater [61], and in surface water affected by effluents from municipal wastewater treatment plants [34]. GIV-FRNAPH showed the lowest resistance to higher temperatures, pH levels, and ultraviolet irradiation [60,62], indicating that GIV-FRNAPH is relatively easily inactivated in the environment. Stronger environmental stresses during the warm months were likely to result in the higher infectivity index of GIV-FRNAPH. Such a trend was not clearly observed for the other genogroups in this study. The infectivity index of GII-FRNAPH, which was suggested to be specific to human fecal contamination, did not show a clear seasonal trend. This might be due to fluctuation in operating conditions in municipal wastewater treatment plants affecting the sites. A previous study has shown GI- and GII-FRNAPH tend to be more inactivated in the warm season than in the cool season in lake environments [35]. Environmental stress and residence time are considered important factors affecting the infectivity index. The relationship between these factors and the infectivity index needs to be clarified in the future.

Comparing the infectivity index of several genogroups of FRNAPHs or viruses may provide further insight into viral inactivation in the natural environment and by water treatments.

### Comparing indicators of fecal contamination and viral inactivation

In this study, HF183, GII-FRNAPH-gene, and crAssphage showed specificity to human feces. At J, the positive rates of these three were almost 100%. The geometric mean concentrations of crAssphage (10^3.6^ copies/100 mL) and HF183 (10^3.0^ copies/100 mL) were almost comparable, but that of GII-FRNAPH-gene (10^1.6^ copies/100 mL) was low, implying that crAssphage and HF183 are better indicators of human fecal contamination than GII-FRNAPH-gene.

Pig-2-Bac, GIV-FRNAPH-gene, and GIV-FRNAPH-inf indicated host specificity to pig feces. At O2, the positive rates of Pig-2-Bac and GIV-FRNAPH-gene were 100%, while that of GIV-FRNAPH-inf was lower (73%). The geometric mean concentration of Pig-2-Bac (10^4.4^ copies/100 mL) was 2 log_10_ higher than that of GIV-FRNAPH-gene (10^2.3^ copies/100 mL). These results imply that Pig-2-Bac is a better indicator of pig fecal contamination. However, GII- and GIV-FRNAPH can be useful tools to estimate the infectivity index of viruses.

### Limitations of the study

Our PCA explained < 60% of the total information. In addition to the presence of the third group of microbes discussed above, low detection frequencies of GIV-FRNAPH at sites other than O2 might contribute to the result. Increasing the concentration factor is the most typical way to improve the detection frequency. However, considering that the observed concentration of Pig-2-Bac at O2 is > 2 log_10_ higher than that at other sites, the required concentration factor can be unrealistically high. Similarly, GII-FRNAPH was detected in low frequencies at O2 and O3 (14% and 27%, respectively). PCA was also performed without these two microbes (S3 Fig), but still only 59.8% of the total information could be explained and positions of the plots remained almost unchanged. These suggest that the low detection frequencies of them was not a significant cause of the result. Additionally, although the PCA could separate microbes associated with pig and human fecal contaminations, it remains unclear that what were influenced by PC1 and PC2. GIV-FRNAPH-inf was detected more frequently during the cool months, while GIV-FRNAPH-gene did not show such a clear seasonality. In our PCA, these two targets were plotted close together. This indicates that the seasonal factor was influenced by the PCA. The cluster analysis was also performed. In the analysis, FPH subgroups formed one large cluster and the other microbes formed another large cluster, indicating that the cluster analysis was not effective in evaluating host specificity of microbes in our samples (S4 Fig).

## Conclusions

In this study, we aimed to evaluate the applicability of FPH subgroups, PMMoV, and crAssphage as indicators of fecal contamination. The host specificity of the FPH subgroups, PMMoV, and crAssphage was evaluated by PCA. We also evaluated the applicability of FRNAPH genogroups as an indicator of viral inactivation by comparing their concentrations measured by cultural and molecular assays.

PCA indicated that GIV-FRNAPH-gene and GIV-FRNAPH-inf were specific to pig feces. PCA also indicated that GII-FRNAPH-gene and crAssphage were specific to human feces. However, PMMoV, GI-FRNAPH-inf, and FDNAPH were suggested not to be specific to either human or pig feces.

The infectivity index indicated that GIV-FRNAPH was highly inactivated during the warm months (July to November). Comparing the infectivity index of several FRNAPH genogroups or viruses may provide further insight into viral inactivation in the natural environment and by water treatments.

## Supporting information

S1 Fig Infectivity index of GII- and GIV-FRNAPHs in the surface water samples at each site. Infectivity index of GII- (A, B, C, and D) and GIV-FRNAPHs (E) in the surface water samples at each site, where the target FRNAPH subgroup showed detection rates of 30% or higher. Infectivity index (Inf. index) was defined as the differences between the log_10_-transformed concentrations of infectious FRNAPHs (MPN) and their gene (copies) as indicated by diagonal lines. The circles represent samples collected during the warm months (from July to November), and the triangles represent samples collected during the cool months (from December to June). “N.D.” on the axis means “not detected.” The white plot on the axis indicates that the sample was negative in cultural and/or RT-qPCR assays.
(TIF)

S2 Fig Principal component analysis (PCA) of observed concentrations of microbial targets excluding those classified in the third group (GI-FRNAPH-inf, GII-FRNAPH-inf, and PMMoV) in the samples (n = 94). The analysis employs concentrations of microbial targets with 24% or higher positive rates excluding those classified in the third group by PCA shown on Fig 3 (i.e., HF183, Pig-2-Bac, GII-FRNAPH-gene, GIV-FRNAPH-gene, GIV-FRNAPH-inf, crAssphage, PMMoV, E. coli, FPH-plaque, FPH-MPN, and FDNAPH). Circles (○) and triangles (Δ) refer to the indicators quantified based on gene and viability, respectively. The vertical and horizontal axes indicate principal components (PC) 1 and 2, which explained 40.0% and 27.5% of the total information, respectively.
(TIF)

S3 Fig Principal component analysis (PCA) of observed concentrations of microbial targets that showed 68% or higher positive rates in the samples (n = 94). The analysis employs concentrations of microbial targets with 68% or higher positive rates (i i.e., HF183, Pig-2-Bac, GI-FRNAPH-inf, GII-FRNAPH-gene, crAssphage, PMMoV, E. coli, FPH-plaque, FPH-MPN, and FDNAPH). Circles (○) and triangles (Δ) refer to the indicators quantified based on gene and viability, respectively. The vertical and horizontal axes indicate principal components (PC) 1 and 2, which explained 34.8% and 25.0% of the total information, respectively.
(TIF)

S4 Fig A dendrogram from the cluster analysis using Ward’s hierarchical method for the observed concentrations of microbial targets (n = 94). (TIF)

S1 Table Primer and TaqMan probe sequences used for (RT-) qPCR and IC-RT-PCR assays in this study. (DOCX)

S2 Table Dates of sample collection. (DOCX)

S3 Table The observed concentrations of microbes (i.e., HF183, BacHum, Pig-2-Bac, GI-, GII- aND GIV- FRNAPH-inf, GII- aND GIV-FRNAPH-gene, crAssphage, PMMoV, E. coli, FPH-plaque, FPH-MPN, FDNAPH) in each sample. (DOCX)

S4 Table MIQE guideline essential information checklist. (DOCX)
