# Supplementary figures and images for: Applicability of F-specific bacteriophage subgroups, PMMoV and crAssphage as indicators of source specific fecal contamination and viral inactivation in rivers in Japan
Source: PLoS One. 2023 Jul 14;18(7):e0288454. doi: 10.1371/journal.pone.0288454 (PMC10348522; doi:10.1371/journal.pone.0288454)

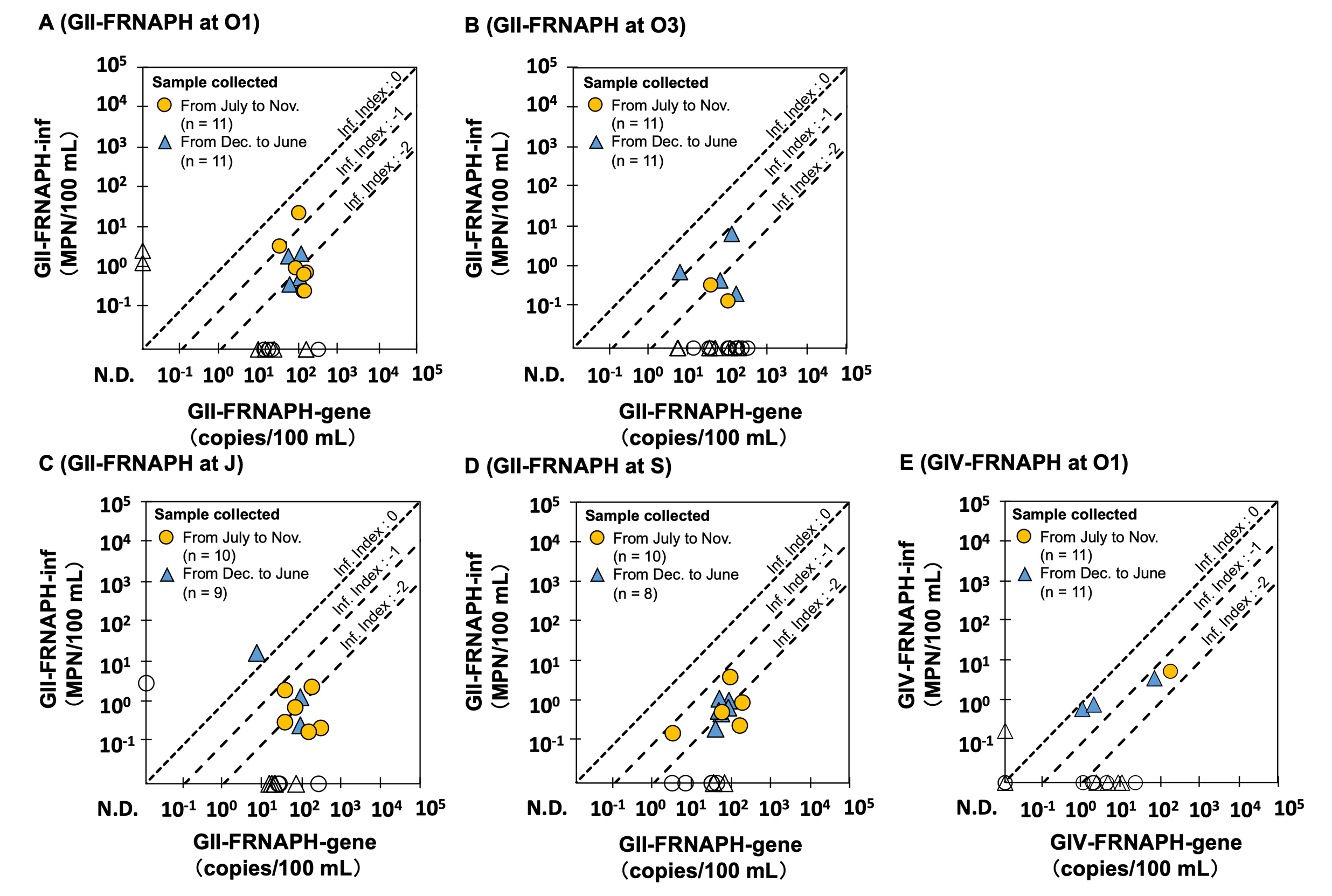

Supplement: S1 Fig — Infectivity index of GII- (A, B, C, and D) and GIV-FRNAPHs (E) in the surface water samples at each site, where the target FRNAPH subgroup showed detection rates of 30% or higher. Infectivity index (Inf. index) was defined as the differences between the log10-transformed concentrations of infectious FRNAPHs (MPN) and their gene (copies) as indicated by diagonal lines. The circles represent samples collected during the warm months (from July to November), and the triangles represent samples collected during the cool months (from December to June). “N.D.” on the axis means “not detected.” The white plot on the axis indicates that the sample was negative in cultural and/or RT-qPCR assays. (TIF) [file pone.0288454.s001.tif]

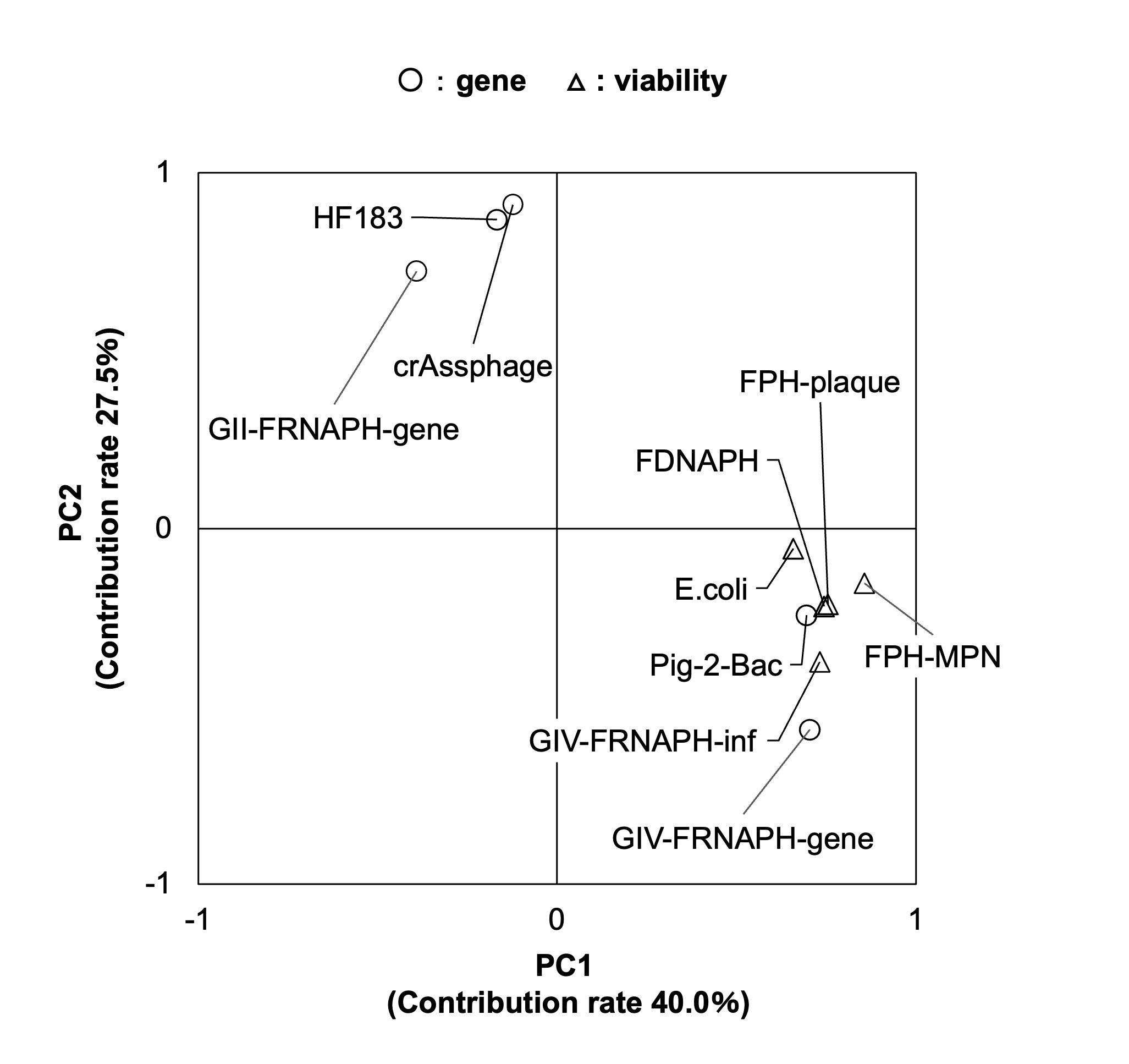

Supplement: S2 Fig — The analysis employs concentrations of microbial targets with 24% or higher positive rates excluding those classified in the third group by PCA shown on Fig 3 (i.e., HF183, Pig-2-Bac, GII-FRNAPH-gene, GIV-FRNAPH-gene, GIV-FRNAPH-inf, crAssphage, PMMoV, E. coli, FPH-plaque, FPH-MPN, and FDNAPH). Circles (○) and triangles (Δ) refer to the indicators quantified based on gene and viability, respectively. The vertical and horizontal axes indicate principal components (PC) 1 and 2, which explained 40.0% and 27.5% of the total information, respectively. (TIF) [file pone.0288454.s002.tif]

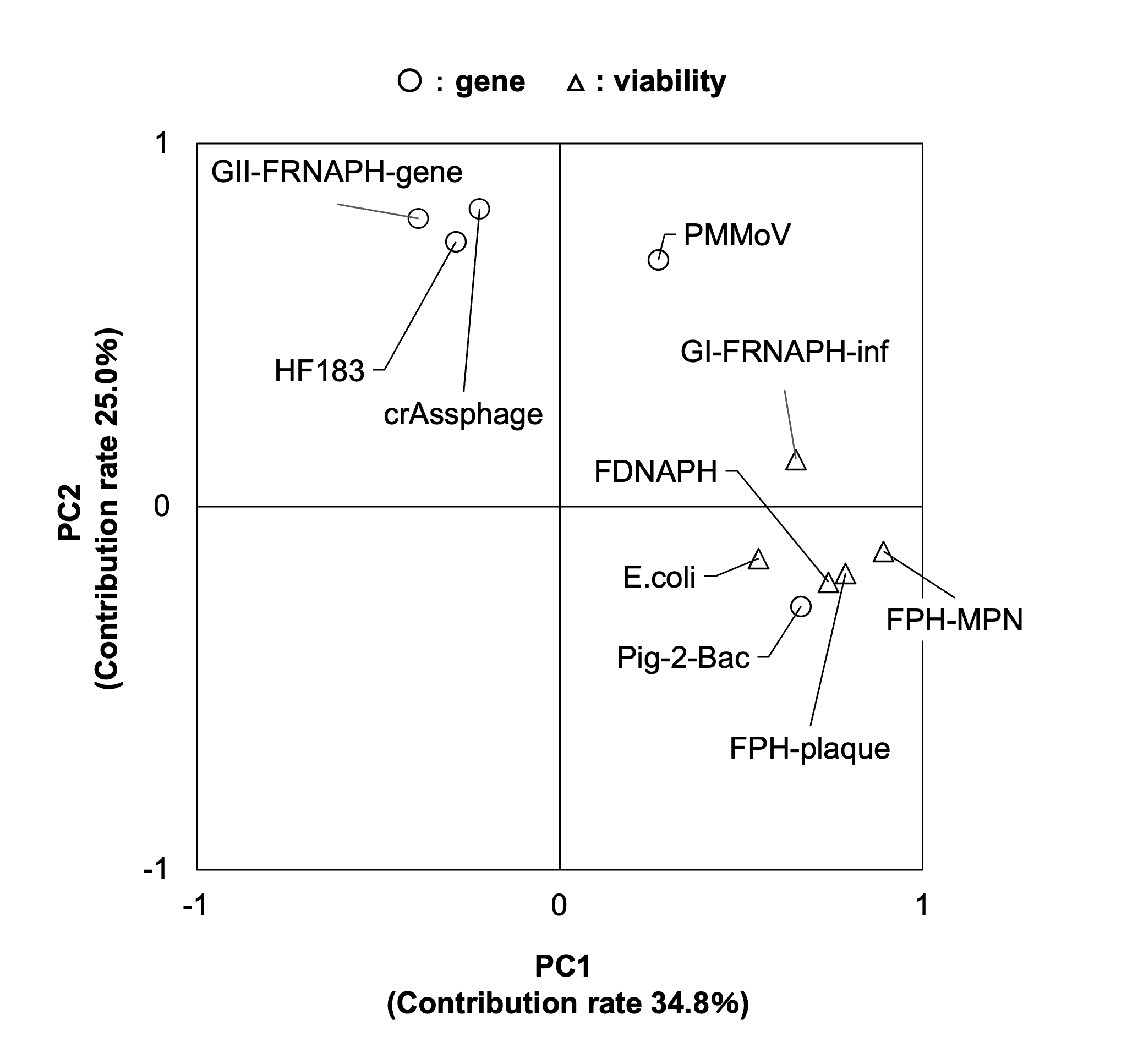

Supplement: S3 Fig — The analysis employs concentrations of microbial targets with 68% or higher positive rates (i i.e., HF183, Pig-2-Bac, GI-FRNAPH-inf, GII-FRNAPH-gene, crAssphage, PMMoV, E. coli, FPH-plaque, FPH-MPN, and FDNAPH). Circles (○) and triangles (Δ) refer to the indicators quantified based on gene and viability, respectively. The vertical and horizontal axes indicate principal components (PC) 1 and 2, which explained 34.8% and 25.0% of the total information, respectively. (TIF) [file pone.0288454.s003.tif]

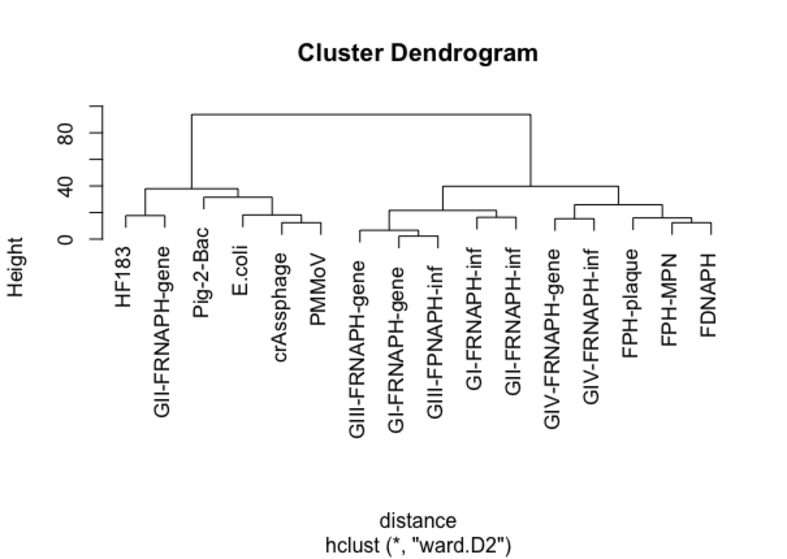

Supplement: S4 Fig — (TIF) [file pone.0288454.s004.tif]
