# Supplementary material for: Applicability of F-specific bacteriophage subgroups, PMMoV and crAssphage as indicators of source specific fecal contamination and viral inactivation in rivers in Japan
Source: PLoS One. 2023 Jul 14;18(7):e0288454. doi: 10.1371/journal.pone.0288454 (PMC10348522; doi:10.1371/journal.pone.0288454)
Supplement: S1 Table — (DOCX) [file pone.0288454.s005.docx]

**S1 Table. Primer and TaqMan probe sequences used for (RT-) qPCR and IC-RT-PCR assay in this study.**

| **Assay** | **Function** | **Sequence (5' → 3')** | **temperature for annealing and extension (°C)** | **Reference** |
| --- | --- | --- | --- | --- |
| Murine Norovirus | Forward primer | CGGTGAAGTGCTTCTGAGGTT | 56 | [54] |
|  | Reverse primer | GCAGCGTCAGTGCTGTCAA |  |  |
|  | Probe | FAM-CGAACCTACATGCGTCAG-MGB-NFQ※ |  |  |
| φX174 phage | Forward primer | ACAAAGTTTGGATTGCTACTGACC | 60 | [55] |
|  | Reverse primer | ﻿CGGCAGCAATAAACTCAACAGG |  |  |
|  | Probe | HEX-﻿CTCTCGTGCTCGTCGCTGCGTTGA-TAMRA |  |  |
| HF183 | Forward primer | ATCATGAGTTCACATGTCCG | 56 | [42] |
|  | Reverse primer | CTTCCTCTCAGAACCCCTATCC |  |  |
|  | Probe | FAM-CTAATGGAACGCATCCC-MGB |  |  |
| BacHum | Forward primer | TGAGTTCACATGTCCGCATGA | 60 | [47] |
|  | Reverse primer | CGTTACCCCGCCTACTATCTAATG |  |  |
|  | Probe | FAM-TCCGGTAGACGATGGGGATGCGTT-TAMRA |  |  |
| Pig-2-Bac | Forward primer | GCATGAATTTAGCTTGCTAAATTTGAT | 60 | [15] |
|  | Reverse primer | ACCTCATACGGTATTAATCCGC |  |  |
|  | Probe | FAM-TCCACGGGATAGCC-MGB |  |  |
| GI-FRNAPH (MS2) | Forward primer | GTCCTGCTCRACTTCCTGT | 59 | [33] |
|  | Reverse primer | ATGGAATTSCGGCTACCTACA |  |  |
|  | Probe | FAM-CGAGACGCTACCWTGGCTATCGC-MGB-NFQ |  |  |
| GII-FRNAPH (GA) | Forward primer | ACCTATGTTCCGATTCASAGAG | 59 | [33] |
|  | Reverse primer | GGTAGGCAAGTCCATCAAAGT |  |  |
|  | Probe | FAM-CACTCGCGATTGTGCTGTCCGATT-MGB-NFQ |  |  |
| GIII-FRNAPH (Qβ) | Forward primer | TTTGAGGCTRTGTTGCGACA | 59 | [33] |
|  |  | CCGTCCGTTGAGGGTATGTT |  |  |
|  | Reverse primer | CCGTGGSGTACACTCTTG |  |  |
|  |  | CGAGGSGTACACGCTTG |  |  |
|  | Probe | FAM-CGGYCATCCGTCCTTCAAGTTTGC-MGB-NFQ |  |  |
| GIV-FRNAPH (SP) | Forward primer | AAGACWGGTCGGTACAAAGT | 59 | [33] |
|  | Reverse primer | ARCTTCACCTCGGGAAKTC |  |  |
|  | Probe | FAM-CCGGATGAAGGCACTGTCCTGAATC-MGB-NFQ |  |  |
| Pepper mild mottle virus | Forward primer | GAGTGGTTTGACCTTAACGTTTGA | 60 | [56] |
|  | Reverse primer | TTGTCGGTTGCAATGCAAGT |  |  |
|  | Probe | FAM-CCTACCGAAGCAAATG-MGB-NFQ |  |  |
| crAssphage | Forward primer | CAGAAGTACAAACTCCTAAAAAACGTAGAG | 60 | [57] |
|  | Reverse primer | GATGACCAATAAACAAGCCATTAGC |  |  |
|  | Probe | FAM-AATAACGATTTACGTGATGTAAC-MGB |  |  |
| GI-Norovirus | Forward primer | CGYTGGATGCGNTTYCATGA | 56 | [58] |
|  | Reverse primer | CTTAGACGCCATCATCATTYAC |  |  |
|  | Probe | FAM-AGATYGCGATCYCCTGTCCA-TAMRA |  |  |
|  |  | FAM-AGATCGCGGTCTCCTGTCCA-TAMRA |  |  |
| GII-Norovirus | Forward primer | CARGARBCNATGTTYAGRTGGATGAG | 56 | [58] |
|  | Reverse primer | TCGACGCCATCTTCATTCACA |  |  |
|  | Probe | FAM-TGGGAGGGCGATCGCAATCT-TAMRA |  |  |

*^a^*: 6-carboxyfluorescein

*^b^*: minor groove binder

*^c^*: nonfluorescent quencher

*^d^*: hexachlorofluorescein

*^e^*: 6-carboxytetrametylrhofdammin
