# Supplementary material for: Applicability of F-specific bacteriophage subgroups, PMMoV and crAssphage as indicators of source specific fecal contamination and viral inactivation in rivers in Japan
Source: PLoS One. 2023 Jul 14;18(7):e0288454. doi: 10.1371/journal.pone.0288454 (PMC10348522; doi:10.1371/journal.pone.0288454)
Supplement: S2 Table — (DOCX) [file pone.0288454.s006.docx]

**S2 Table. Dates of sample collection.**

| Year | Month | Day | Sites collected a sample |
| --- | --- | --- | --- |
| 2019 | Nov. | 18 | O1, O2, O3 |
|  | Dec. | 16 | O1, O2, O3 |
| 2020 | Jan. | 28 | O1, O2, O3 |
|  | Feb. | 20 | O1, O2, O3, J, S |
|  | Mar. | 9 | O1, O2, O3, J, S |
|  | Jun. | 11 | O1, O2, O3, J |
|  | Jul. | 27 | O1, O2, O3 |
|  |  | 29 | J, S |
|  | Aug. | 24 | O1, O2, O3 |
|  | Sep. | 1 | J, S |
|  |  | 17 | O1, O2, O3, J |
|  |  | 19 | S |
|  | Oct. | 12 | O1, O2, O3, J, S |
|  | Nov. | 16 | O1, O2, O3, J, S |
|  | Dec. | 14 | O1, O2, O3, J, S |
| 2021 | Jun. | 18 | O1, O2, O3, S |
|  |  | 29 | J |
|  | Feb. | 26 | O1, O2, O3, J, S |
|  | Mar. | 25 | O1, O2, O3, J, S |
|  | Apr. | 16 | O1, O2, O3, J, S |
|  | May | 28 | O1, O2, O3, J, S |
|  | Jun. | 16 | O1, O2, O3, J, S |
|  | Jul. | 30 | O1, O2, O3, J, S |
|  | Aug. | 20 | O1, O2, O3, J, S |
|  | Sep. | 24 | O1, O2, O3, J, S |
|  | Oct. | 25 | O1, O2, O3, J, S |
|  | Nov. | 22 | O1, O2, O3, J, S |
