# Supplementary material for: Applicability of F-specific bacteriophage subgroups, PMMoV and crAssphage as indicators of source specific fecal contamination and viral inactivation in rivers in Japan
Source: PLoS One. 2023 Jul 14;18(7):e0288454. doi: 10.1371/journal.pone.0288454 (PMC10348522; doi:10.1371/journal.pone.0288454)
Supplement: S3 Table — (DOCX) [file pone.0288454.s007.docx]

**S3 Table.** The concentrations of microbes (i.e., HF183, BacHum, Pig-2-Bac, GI-, GII- and GIV- FRNAPH-inf, GII- and GIV-FRNAPH-gene, crAssphage, PMMoV, *E. coli*, FPH-plaque, FPH-MPN, FDNAPH).

| Site | Year | Month | HF183 | BacHum | Pig-2-Bac | GI-FRNAPH-gene | GII-FRNAPH-gene | GIII-FRNAPH-gene | GIV-FRNAPH-gene | GI-FRNAPH-Inf | GII-FRNAPH-Inf | GIII-FRNAPH-Inf | GIV-FRNAPH-Inf | crAssphage | PMMoV | GI-Norovirus | GII-Norovirus | E.coli | FPH-MPN | FPH-plaque | FDNAPH |
| --- | --- | --- | --- | --- | --- | --- | --- | --- | --- | --- | --- | --- | --- | --- | --- | --- | --- | --- | --- | --- | --- |
| O1 | 2019 | 11 | N.D. | 1.9 | 2.9 | N.D. | 1.9 | N.D. | 2.2 | 0.3 | 0.0 | N.D. | 0.8 | 3.1 | 4.4 | N.D. | N.D. | 2.6 | 1.8 | 2.2 | -1.1 |
| O1 | 2019 | 12 | 0.9 | N.D. | 2.3 | N.D. | 1.8 | N.D. | 0.3 | 0.3 | -0.4 | N.D. | -0.1 | 3.1 | 4.1 | N.D. | N.D. | 1.9 | 0.3 | 0.3 | -0.1 |
| O1 | 2020 | 1 | 2.5 | N.D. | 3.6 | N.D. | 2.1 | N.D. | 0.0 | 0.1 | 0.4 | N.D. | -0.2 | 3.1 | 4.2 | N.D. | N.D. | 2.0 | 1.2 | 0.8 | 0.1 |
| O1 | 2020 | 2 | 1.5 | 2.6 | 3.0 | N.D. | 2.2 | N.D. | N.D. | 0.4 | N.D. | N.D. | -0.7 | 3.5 | 4.0 | N.D. | N.D. | 2.2 | 0.4 | 0.3 | - |
| O1 | 2020 | 3 | 2.3 | 2.3 | 4.0 | N.D. | 2.2 | 0.4 | 0.6 | 0.0 | N.D. | N.D. | N.D. | 3.4 | 4.3 | N.D. | N.D. | 1.7 | 1.1 | 0.3 | - |
| O1 | 2020 | 6 | 2.2 | N.D. | 2.0 | N.D. | N.D. | N.D. | N.D. | N.D. | 0.2 | N.D. | N.D. | 3.7 | 4.3 | N.D. | N.D. | 1.9 | -0.4 | 0.3 | - |
| O1 | 2020 | 7 | 2.3 | 2.7 | 3.3 | N.D. | 2.2 | N.D. | 1.3 | -0.6 | -0.1 | N.D. | N.D. | 3.0 | 4.1 | N.D. | N.D. | 2.4 | 0.9 | 0.3 | 0.1 |
| O1 | 2020 | 8 | 1.8 | 2.1 | N.D. | N.D. | 2.5 | N.D. | N.D. | 0.1 | N.D. | N.D. | N.D. | 3.5 | 4.5 | N.D. | N.D. | 1.7 | 0.7 | 0.3 | 0.0 |
| O1 | 2020 | 9 | 2.4 | 2.3 | 1.6 | N.D. | 2.1 | N.D. | 0.2 | 0.7 | -0.6 | N.D. | N.D. | 3.3 | 4.0 | N.D. | N.D. | 2.3 | 1.0 | 0.6 | -0.1 |
| O1 | 2020 | 10 | 1.7 | 3.0 | 1.9 | N.D. | 2.1 | 0.5 | N.D. | -0.3 | -0.6 | N.D. | N.D. | 1.8 | 4.3 | N.D. | N.D. | 2.5 | 0.7 | N.D. | 0.3 |
| O1 | 2020 | 11 | 2.5 | 2.5 | 2.5 | N.D. | 2.1 | N.D. | 0.6 | -0.4 | -0.1 | N.D. | N.D. | 3.6 | 4.5 | N.D. | N.D. | 2.4 | 0.3 | N.D. | 0.3 |
| O1 | 2020 | 12 | 1.8 | - | 3.8 | N.D. | N.D. | N.D. | 0.3 | 0.4 | 0.4 | N.D. | N.D. | 2.6 | 3.1 | N.D. | N.D. | 2.6 | 1.3 | 1.2 | 0.2 |
| O1 | 2021 | 1 | 2.4 | - | 4.4 | N.D. | 1.1 | N.D. | 1.9 | -0.4 | N.D. | N.D. | 0.6 | 3.5 | 3.1 | N.D. | N.D. | 2.2 | 0.6 | 1.1 | 0.6 |
| O1 | 2021 | 2 | 2.4 | - | 4.7 | N.D. | 1.0 | N.D. | 0.9 | 0.3 | N.D. | N.D. | N.D. | 3.6 | 3.2 | N.D. | N.D. | 2.0 | 0.8 | 0.6 | 0.8 |
| O1 | 2021 | 3 | 2.8 | - | 4.4 | N.D. | 1.4 | N.D. | 1.0 | 0.0 | N.D. | N.D. | N.D. | 3.6 | 3.0 | N.D. | N.D. | 2.0 | 2.5 | 0.3 | 0.0 |
| O1 | 2021 | 4 | 1.9 | - | 3.3 | N.D. | 2.0 | N.D. | N.D. | 0.3 | -0.3 | N.D. | N.D. | 3.7 | 3.6 | N.D. | N.D. | 1.6 | 1.0 | 1.1 | 0.7 |
| O1 | 2021 | 5 | 2.7 | - | 3.3 | N.D. | 1.7 | N.D. | N.D. | N.D. | 0.3 | N.D. | N.D. | 4.0 | 3.3 | N.D. | N.D. | 1.8 | 2.3 | 0.3 | 0.3 |
| O1 | 2021 | 6 | 2.4 | - | 3.1 | N.D. | 0.7 | N.D. | 0.7 | - | - | - | - | 3.8 | 2.9 | N.D. | N.D. | 2.3 | 1.5 | 0.3 | -0.3 |
| O1 | 2021 | 7 | 2.2 | - | N.D. | N.D. | 1.1 | N.D. | 0.0 | -1.2 | N.D. | N.D. | N.D. | 3.7 | 3.2 | N.D. | N.D. | 1.9 | 0.1 | N.D. | -0.4 |
| O1 | 2021 | 8 | 2.0 | - | 2.1 | N.D. | 1.2 | N.D. | N.D. | N.D. | N.D. | N.D. | N.D. | 3.0 | 2.5 | N.D. | N.D. | 2.1 | -0.4 | N.D. | -0.4 |
| O1 | 2021 | 9 | 1.9 | - | 2.4 | N.D. | 1.3 | N.D. | 0.3 | -1.2 | N.D. | N.D. | N.D. | 3.3 | 2.9 | N.D. | N.D. | 2.0 | 0.9 | 0.6 | 0.9 |
| O1 | 2021 | 10 | 2.4 | - | 2.1 | N.D. | 1.5 | N.D. | N.D. | -0.8 | 0.6 | N.D. | N.D. | 3.0 | 3.5 | N.D. | N.D. | 2.0 | -0.3 | 0.6 | -1.2 |
| O1 | 2021 | 11 | 2.7 | - | 2.4 | N.D. | 2.0 | N.D. | N.D. | -1.1 | 1.4 | N.D. | N.D. | 3.7 | 3.9 | N.D. | N.D. | 2.2 | 1.4 | 1.9 | -0.3 |
| O2 | 2019 | 11 | 1.3 | 2.8 | 3.2 | N.D. | 1.2 | N.D. | 2.1 | 0.5 | N.D. | N.D. | N.D. | 2.2 | 5.8 | N.D. | N.D. | 3.3 | 1.6 | 1.8 | -0.5 |
| O2 | 2019 | 12 | N.D. | N.D. | 2.9 | N.D. | 0.3 | N.D. | 1.5 | N.D. | N.D. | N.D. | 0.8 | 1.3 | 4.0 | N.D. | N.D. | 2.4 | 0.8 | 1.4 | 0.1 |
| O2 | 2020 | 1 | N.D. | N.D. | 4.6 | N.D. | N.D. | N.D. | 2.4 | 0.0 | N.D. | N.D. | 0.2 | 1.4 | 3.9 | N.D. | N.D. | 3.6 | 1.5 | 1.9 | 1.5 |
| O2 | 2020 | 2 | N.D. | N.D. | 4.3 | N.D. | N.D. | N.D. | 1.9 | -0.9 | N.D. | N.D. | 0.8 | 2.6 | 4.0 | N.D. | N.D. | 3.1 | 1.2 | 1.3 | - |
| O2 | 2020 | 3 | N.D. | N.D. | 4.9 | N.D. | N.D. | N.D. | 2.3 | N.D. | N.D. | N.D. | 1.2 | 1.9 | 3.7 | N.D. | N.D. | 3.3 | 1.9 | 2.0 | - |
| O2 | 2020 | 6 | N.D. | N.D. | 4.1 | N.D. | N.D. | N.D. | 2.3 | -0.8 | N.D. | N.D. | 0.2 | 2.1 | 3.1 | N.D. | N.D. | 2.9 | 1.3 | 1.7 | - |
| O2 | 2020 | 7 | 1.3 | 1.9 | 5.5 | N.D. | 1.0 | 0.7 | 3.2 | N.D. | 0.1 | 0.2 | 1.6 | 2.4 | 3.6 | N.D. | N.D. | 4.1 | 2.0 | 2.7 | 2.0 |
| O2 | 2020 | 8 | N.D. | 1.8 | 4.3 | N.D. | 1.0 | N.D. | 1.9 | 2.0 | N.D. | N.D. | 0.9 | 2.2 | 4.2 | N.D. | N.D. | 2.3 | 2.6 | 2.0 | 2.6 |
| O2 | 2020 | 9 | 2.0 | 1.4 | 4.7 | N.D. | N.D. | N.D. | 0.9 | 0.8 | 0.0 | N.D. | N.D. | 1.6 | 3.5 | N.D. | N.D. | 3.0 | 0.8 | 0.8 | 0.8 |
| O2 | 2020 | 10 | N.D. | N.D. | 4.0 | N.D. | N.D. | N.D. | 1.6 | 0.9 | N.D. | N.D. | N.D. | 3.0 | 3.0 | N.D. | N.D. | 3.1 | 2.6 | 2.4 | 2.6 |
| O2 | 2020 | 11 | N.D. | N.D. | 3.3 | N.D. | 0.3 | N.D. | 2.3 | -1.0 | 1.4 | N.D. | 1.4 | 1.7 | 3.9 | N.D. | N.D. | 2.3 | 2.1 | 1.1 | 2.1 |
| O2 | 2020 | 12 | 1.9 | - | 5.8 | N.D. | 0.0 | N.D. | 3.4 | 0.5 | N.D. | N.D. | 2.2 | 2.4 | 3.3 | N.D. | N.D. | 3.5 | 3.2 | 2.1 | 1.5 |
| O2 | 2021 | 1 | 1.0 | - | 5.9 | N.D. | N.D. | N.D. | 3.5 | 2.1 | N.D. | N.D. | 2.4 | 2.0 | 2.5 | N.D. | N.D. | 3.5 | 3.4 | 2.7 | 1.4 |
| O2 | 2021 | 2 | 1.4 | - | 6.8 | N.D. | N.D. | N.D. | 4.2 | N.D. | N.D. | N.D. | 2.8 | 2.4 | 3.5 | N.D. | N.D. | 4.2 | 3.8 | 3.2 | 2.8 |
| O2 | 2021 | 3 | N.D. | - | 5.7 | N.D. | N.D. | N.D. | 2.9 | 0.6 | N.D. | N.D. | 1.9 | 1.8 | 3.1 | N.D. | N.D. | 3.3 | 2.9 | 1.9 | 1.6 |
| O2 | 2021 | 4 | N.D. | - | 4.9 | N.D. | N.D. | N.D. | 3.2 | -0.4 | N.D. | N.D. | 1.3 | 2.6 | 4.0 | N.D. | N.D. | 2.3 | 3.1 | 1.3 | 1.7 |
| O2 | 2021 | 5 | 1.3 | - | 4.3 | N.D. | N.D. | N.D. | 3.3 | N.D. | N.D. | N.D. | 1.1 | 2.1 | 3.7 | N.D. | N.D. | 2.4 | 1.7 | 1.3 | 1.3 |
| O2 | 2021 | 6 | N.D. | - | 4.3 | N.D. | 0.6 | N.D. | 2.8 | - | - | - | - | 3.1 | 3.3 | N.D. | N.D. | 2.2 | 2.8 | 1.5 | 0.4 |
| O2 | 2021 | 7 | 0.7 | - | 3.3 | N.D. | N.D. | N.D. | 2.0 | 2.4 | N.D. | N.D. | N.D. | 1.3 | 3.3 | N.D. | N.D. | 2.1 | 3.4 | 2.3 | 0.5 |
| O2 | 2021 | 8 | N.D. | - | 4.6 | N.D. | N.D. | N.D. | 2.4 | -0.4 | N.D. | N.D. | 0.0 | N.D. | 2.8 | N.D. | N.D. | 2.8 | 0.4 | 0.9 | -0.7 |
| O2 | 2021 | 9 | N.D. | - | 4.2 | N.D. | N.D. | N.D. | 2.2 | -0.9 | N.D. | N.D. | -0.2 | 1.3 | 4.1 | N.D. | N.D. | 2.4 | 1.3 | 1.3 | 0.9 |
| O2 | 2021 | 10 | N.D. | - | 3.2 | N.D. | N.D. | N.D. | 1.0 | -0.7 | N.D. | N.D. | N.D. | 0.6 | 2.9 | N.D. | N.D. | 2.1 | 0.7 | 1.4 | 0.7 |
| O2 | 2021 | 11 | N.D. | - | 3.1 | N.D. | N.D. | N.D. | 1.3 | -0.4 | N.D. | N.D. | N.D. | 1.4 | 3.4 | N.D. | N.D. | 1.7 | 0.8 | 0.6 | 0.8 |
| O3 | 2019 | 11 | 0.5 | 3.4 | 1.7 | N.D. | 1.5 | N.D. | N.D. | -0.1 | -0.4 | -1.8 | N.D. | 2.2 | 3.6 | N.D. | N.D. | 2.0 | 0.6 | 0.3 | -0.8 |
| O3 | 2019 | 12 | 1.3 | N.D. | 1.7 | N.D. | 2.2 | N.D. | 1.4 | 0.9 | -0.6 | N.D. | 0.2 | 3.5 | 4.2 | N.D. | N.D. | 1.4 | 0.6 | 1.0 | 0.2 |
| O3 | 2020 | 1 | 2.5 | 2.1 | 3.3 | N.D. | 1.8 | N.D. | N.D. | 1.2 | -0.3 | N.D. | -1.2 | 3.0 | 4.4 | N.D. | N.D. | 1.8 | 1.6 | 1.7 | -0.2 |
| O3 | 2020 | 2 | N.D. | 1.7 | 2.2 | N.D. | 1.5 | N.D. | N.D. | 0.0 | N.D. | N.D. | N.D. | 2.5 | 3.8 | N.D. | N.D. | 1.8 | 0.0 | 0.3 | - |
| O3 | 2020 | 3 | 2.6 | 1.8 | 3.1 | N.D. | 2.2 | N.D. | N.D. | -0.1 | N.D. | N.D. | N.D. | 3.5 | 4.0 | N.D. | N.D. | 1.1 | 0.5 | N.D. | - |
| O3 | 2020 | 6 | 2.8 | 2.0 | 2.7 | N.D. | 2.0 | N.D. | N.D. | -0.6 | N.D. | N.D. | N.D. | 3.9 | 4.2 | N.D. | N.D. | 1.8 | -0.5 | 0.9 | - |
| O3 | 2020 | 7 | N.D. | 1.6 | 2.2 | N.D. | 2.5 | N.D. | N.D. | 0.0 | N.D. | N.D. | N.D. | 2.8 | 4.4 | N.D. | N.D. | 2.5 | 0.0 | N.D. | -0.2 |
| O3 | 2020 | 8 | 1.8 | 1.9 | N.D. | N.D. | 2.3 | N.D. | N.D. | N.D. | N.D. | N.D. | N.D. | 3.0 | 4.1 | N.D. | N.D. | 2.0 | -0.3 | N.D. | -0.3 |
| O3 | 2020 | 9 | 2.4 | 2.1 | 2.4 | N.D. | 2.2 | N.D. | N.D. | N.D. | N.D. | N.D. | N.D. | 3.3 | 3.6 | N.D. | N.D. | 2.1 | -0.2 | N.D. | -0.2 |
| O3 | 2020 | 10 | 2.2 | 2.3 | 2.4 | N.D. | 2.0 | N.D. | N.D. | 0.3 | N.D. | N.D. | N.D. | 3.6 | 3.6 | N.D. | N.D. | 2.4 | 0.5 | 1.5 | -0.8 |
| O3 | 2020 | 11 | 2.0 | 2.2 | 3.6 | N.D. | 1.5 | N.D. | 0.3 | N.D. | N.D. | N.D. | N.D. | 2.4 | 3.8 | N.D. | N.D. | 2.1 | -0.7 | N.D. | -0.7 |
| O3 | 2020 | 12 | 2.4 | - | 3.1 | N.D. | 0.7 | N.D. | N.D. | N.D. | -0.1 | N.D. | N.D. | 3.3 | 3.5 | N.D. | N.D. | 2.2 | 0.0 | N.D. | -0.6 |
| O3 | 2021 | 1 | 2.3 | - | 3.9 | N.D. | 0.7 | N.D. | N.D. | -0.2 | N.D. | N.D. | N.D. | 3.0 | 3.6 | N.D. | N.D. | 1.6 | 0.8 | N.D. | -0.2 |
| O3 | 2021 | 2 | 2.6 | - | 4.2 | N.D. | 0.7 | N.D. | N.D. | -1.1 | N.D. | N.D. | -0.1 | 3.1 | 3.9 | N.D. | N.D. | 1.4 | 1.0 | N.D. | 1.0 |
| O3 | 2021 | 3 | 2.3 | - | 3.1 | N.D. | 1.6 | N.D. | N.D. | -1.1 | N.D. | N.D. | N.D. | 3.8 | 3.6 | N.D. | N.D. | 1.1 | 0.0 | N.D. | 0.0 |
| O3 | 2021 | 4 | 3.6 | - | 2.4 | N.D. | 2.1 | N.D. | N.D. | -0.3 | 0.8 | N.D. | N.D. | 4.2 | 3.8 | N.D. | N.D. | 2.1 | 0.2 | N.D. | 0.1 |
| O3 | 2021 | 5 | 2.3 | - | 3.6 | N.D. | 1.5 | N.D. | N.D. | -1.2 | N.D. | N.D. | N.D. | 3.3 | 3.4 | N.D. | N.D. | 1.4 | -0.7 | 0.6 | -1.2 |
| O3 | 2021 | 6 | 3.0 | - | 2.7 | N.D. | 0.6 | N.D. | N.D. | - | - | - | - | 3.9 | 3.2 | N.D. | N.D. | 2.4 | 1.7 | N.D. | -1.1 |
| O3 | 2021 | 7 | 2.8 | - | 2.1 | N.D. | 2.0 | N.D. | N.D. | N.D. | N.D. | N.D. | N.D. | 2.8 | 3.2 | N.D. | N.D. | 2.3 | -1.2 | N.D. | N.D. |
| O3 | 2021 | 8 | 2.1 | - | 3.6 | N.D. | 1.5 | N.D. | N.D. | N.D. | N.D. | N.D. | N.D. | 2.8 | 2.9 | N.D. | N.D. | 1.8 | -0.2 | N.D. | -0.2 |
| O3 | 2021 | 9 | 1.9 | - | 2.5 | N.D. | 2.2 | N.D. | N.D. | -0.9 | N.D. | N.D. | N.D. | 2.7 | 3.4 | N.D. | N.D. | 2.5 | 0.0 | 0.3 | -0.7 |
| O3 | 2021 | 10 | 2.3 | - | 3.3 | N.D. | 1.1 | N.D. | 0.4 | N.D. | N.D. | N.D. | N.D. | 3.1 | 3.3 | N.D. | N.D. | 1.9 | -0.7 | N.D. | -0.7 |
| O3 | 2021 | 11 | 2.7 | - | 1.9 | N.D. | 2.0 | N.D. | N.D. | N.D. | -0.8 | N.D. | N.D. | 3.5 | 3.6 | N.D. | N.D. | 2.4 | 0.3 | 0.3 | 0.0 |
| J | 2020 | 2 | 2.3 | 3.6 | N.D. | N.D. | 2.0 | N.D. | N.D. | -0.2 | 0.1 | N.D. | N.D. | 3.2 | 4.9 | N.D. | N.D. | 1.8 | 0.8 | 1.1 | - |
| J | 2020 | 3 | 2.5 | 2.6 | 2.5 | N.D. | 1.8 | 1.5 | N.D. | 0.8 | N.D. | N.D. | N.D. | 3.5 | 4.8 | N.D. | N.D. | 1.4 | 0.8 | 0.6 | - |
| J | 2020 | 6 | 2.8 | 2.7 | N.D. | N.D. | 1.3 | N.D. | N.D. | 0.3 | N.D. | N.D. | N.D. | 3.1 | 4.6 | N.D. | N.D. | 1.8 | 0.7 | 1.2 | - |
| J | 2020 | 7 | 2.1 | 2.5 | N.D. | N.D. | N.D. | N.D. | N.D. | -0.4 | 0.5 | N.D. | N.D. | 2.7 | 2.3 | N.D. | N.D. | 2.3 | 0.1 | 0.6 | N.D. |
| J | 2020 | 8 | 3.3 | 3.1 | N.D. | N.D. | 2.5 | 0.4 | N.D. | 0.1 | -0.6 | N.D. | N.D. | 4.0 | 4.8 | N.D. | N.D. | 2.2 | -0.1 | 0.6 | -0.1 |
| J | 2020 | 9 | 3.2 | 2.9 | 2.7 | N.D. | 2.2 | N.D. | 0.0 | 0.7 | -0.7 | N.D. | N.D. | 3.7 | 4.6 | N.D. | N.D. | 2.1 | 0.7 | 0.8 | 0.0 |
| J | 2020 | 10 | 2.9 | 2.9 | 1.5 | N.D. | 2.4 | N.D. | N.D. | 0.0 | N.D. | N.D. | N.D. | 2.6 | 4.7 | N.D. | N.D. | 2.1 | 0.6 | 0.8 | -0.4 |
| J | 2020 | 11 | 2.7 | 2.2 | 1.2 | N.D. | 2.2 | N.D. | N.D. | 0.1 | 0.4 | N.D. | N.D. | 3.5 | 4.5 | N.D. | N.D. | 1.9 | 1.6 | N.D. | 1.3 |
| J | 2020 | 12 | 2.9 | - | 2.4 | N.D. | 1.2 | N.D. | N.D. | N.D. | -0.2 | N.D. | N.D. | 3.5 | 4.1 | N.D. | N.D. | 2.3 | 0.4 | N.D. | 0.4 |
| J | 2021 | 1 | 2.7 | - | 2.3 | N.D. | 1.3 | N.D. | N.D. | 1.3 | N.D. | N.D. | N.D. | 3.9 | 3.9 | N.D. | N.D. | 2.0 | 1.4 | 0.3 | -0.8 |
| J | 2021 | 2 | 4.4 | - | 3.4 | N.D. | 1.9 | N.D. | N.D. | -0.6 | -0.6 | N.D. | N.D. | 5.8 | 5.3 | N.D. | N.D. | 2.4 | 0.9 | 0.3 | -0.3 |
| J | 2021 | 3 | 2.7 | - | 3.0 | N.D. | 1.1 | N.D. | N.D. | -0.9 | N.D. | N.D. | N.D. | 3.1 | 3.6 | N.D. | N.D. | 1.7 | 0.3 | N.D. | 0.3 |
| J | 2021 | 4 | 2.9 | - | 2.2 | N.D. | 1.2 | N.D. | N.D. | -0.2 | N.D. | N.D. | N.D. | 4.0 | 4.1 | N.D. | N.D. | 1.5 | 0.6 | N.D. | 0.6 |
| J | 2021 | 5 | 3.7 | - | 3.0 | N.D. | 0.8 | N.D. | N.D. | -0.5 | 1.2 | N.D. | N.D. | 4.3 | 4.2 | N.D. | N.D. | 2.1 | 1.2 | 1.1 | -0.9 |
| J | 2021 | 6 | 3.1 | - | 2.3 | N.D. | 0.7 | N.D. | N.D. | - | - | - | - | 4.2 | 3.9 | N.D. | N.D. | 2.0 | 1.5 | N.D. | N.D. |
| J | 2021 | 7 | 3.5 | - | 8.0 | N.D. | 1.6 | N.D. | N.D. | -0.1 | 0.3 | N.D. | N.D. | 3.9 | 4.1 | N.D. | N.D. | 2.5 | 0.7 | 0.6 | 0.0 |
| J | 2021 | 8 | 3.4 | - | 1.6 | N.D. | 1.5 | N.D. | N.D. | 0.2 | -0.5 | N.D. | N.D. | 3.6 | 3.9 | N.D. | N.D. | 3.0 | 0.5 | 1.1 | 0.5 |
| J | 2021 | 9 | 3.2 | - | 2.0 | N.D. | 1.4 | N.D. | N.D. | -1.2 | N.D. | N.D. | N.D. | 3.5 | 3.9 | N.D. | N.D. | 1.9 | -0.4 | 0.3 | -0.8 |
| J | 2021 | 10 | 2.9 | - | 1.8 | N.D. | 1.8 | N.D. | N.D. | -0.1 | -0.1 | N.D. | N.D. | 3.2 | 3.9 | N.D. | N.D. | 1.6 | -0.1 | N.D. | -0.8 |
| J | 2021 | 11 | 2.8 | - | 1.1 | N.D. | 1.4 | N.D. | N.D. | N.D. | N.D. | N.D. | N.D. | 3.4 | 3.8 | N.D. | N.D. | 2.0 | -1.0 | 0.6 | N.D. |
| S | 2020 | 2 | 2.2 | 3.3 | N.D. | N.D. | 1.9 | N.D. | N.D. | 0.5 | 0.0 | N.D. | N.D. | 3.8 | 4.1 | N.D. | N.D. | 2.2 | 0.7 | 0.3 | - |
| S | 2020 | 3 | 2.1 | 2.6 | N.D. | N.D. | 1.8 | N.D. | N.D. | -0.6 | N.D. | N.D. | N.D. | 3.6 | 3.9 | N.D. | N.D. | 2.2 | 0.6 | N.D. | - |
| S | 2020 | 7 | 2.6 | 2.9 | N.D. | N.D. | 2.0 | N.D. | N.D. | -0.8 | 0.6 | N.D. | N.D. | 3.5 | 3.8 | N.D. | N.D. | 2.1 | 2.0 | N.D. | 0.3 |
| S | 2020 | 8 | 3.6 | 3.2 | N.D. | N.D. | 2.3 | N.D. | N.D. | N.D. | 0.0 | N.D. | N.D. | 4.2 | 4.5 | N.D. | N.D. | 3.0 | 0.4 | N.D. | 0.4 |
| S | 2020 | 9 | N.D. | N.D. | N.D. | N.D. | 1.5 | N.D. | N.D. | N.D. | N.D. | N.D. | N.D. | 2.6 | 3.5 | N.D. | N.D. | 1.8 | 1.4 | 0.9 | 1.4 |
| S | 2020 | 10 | N.D. | 1.1 | N.D. | N.D. | 0.5 | N.D. | N.D. | N.D. | N.D. | N.D. | N.D. | 3.4 | 3.0 | N.D. | N.D. | 0.9 | -0.7 | N.D. | -0.7 |
| S | 2020 | 11 | 1.6 | 1.6 | N.D. | N.D. | 1.5 | N.D. | 0.4 | N.D. | N.D. | N.D. | N.D. | 2.4 | 2.9 | N.D. | N.D. | 1.3 | 0.1 | N.D. | 0.1 |
| S | 2020 | 12 | 3.4 | - | 2.4 | N.D. | 1.9 | N.D. | N.D. | N.D. | -0.1 | N.D. | N.D. | 4.4 | 4.0 | N.D. | N.D. | 3.3 | 1.4 | 0.9 | -0.6 |
| S | 2021 | 1 | 2.8 | - | 2.9 | N.D. | 1.7 | N.D. | N.D. | 0.9 | -0.3 | N.D. | -0.3 | 3.7 | 3.4 | N.D. | N.D. | 1.7 | 1.3 | N.D. | 0.1 |
| S | 2021 | 2 | 2.7 | - | 2.4 | N.D. | 1.6 | N.D. | N.D. | -0.2 | -0.7 | N.D. | N.D. | 3.9 | 3.7 | N.D. | N.D. | 2.0 | 0.8 | 0.3 | 0.1 |
| S | 2021 | 3 | 3.5 | - | 3.0 | N.D. | 1.7 | N.D. | N.D. | 1.1 | 0.1 | N.D. | N.D. | 4.9 | 4.1 | N.D. | N.D. | 2.8 | 1.5 | 0.8 | 1.5 |
| S | 2021 | 4 | 2.6 | - | 2.1 | N.D. | 1.7 | N.D. | 1.5 | N.D. | -0.2 | N.D. | N.D. | 4.0 | 3.4 | N.D. | N.D. | 2.4 | 0.2 | 0.8 | -0.1 |
| S | 2021 | 5 | 2.9 | - | 2.6 | N.D. | 1.5 | N.D. | N.D. | N.D. | N.D. | N.D. | N.D. | 4.0 | 3.8 | N.D. | N.D. | 2.8 | -0.1 | 0.8 | -0.9 |
| S | 2021 | 6 | 2.7 | - | N.D. | N.D. | 0.8 | N.D. | N.D. | - | - | - | - | 4.0 | 3.4 | N.D. | N.D. | 3.0 | 1.2 | N.D. | N.D. |
| S | 2021 | 7 | 3.2 | - | N.D. | N.D. | 2.2 | N.D. | N.D. | N.D. | -0.6 | N.D. | N.D. | 4.5 | 3.8 | N.D. | N.D. | 2.7 | -0.4 | 0.3 | -0.4 |
| S | 2021 | 8 | 1.7 | - | 0.7 | N.D. | 0.8 | N.D. | N.D. | N.D. | N.D. | N.D. | N.D. | 2.9 | 2.7 | N.D. | N.D. | 2.5 | -1.3 | N.D. | N.D. |
| S | 2021 | 9 | 2.4 | - | N.D. | N.D. | 0.5 | N.D. | N.D. | -1.2 | -0.8 | N.D. | N.D. | 3.5 | 2.6 | N.D. | N.D. | 3.0 | -0.9 | N.D. | -1.2 |
| S | 2021 | 10 | 2.5 | - | N.D. | N.D. | 1.6 | N.D. | N.D. | N.D. | N.D. | N.D. | N.D. | 3.7 | 3.8 | N.D. | N.D. | 2.3 | -0.9 | N.D. | -0.9 |
| S | 2021 | 11 | 3.0 | - | N.D. | N.D. | 1.7 | N.D. | N.D. | -1.2 | -0.2 | N.D. | N.D. | 4.0 | 3.7 | N.D. | N.D. | 2.3 | -1.2 | N.D. | N.D. |
