# Supplementary material for: Applicability of F-specific bacteriophage subgroups, PMMoV and crAssphage as indicators of source specific fecal contamination and viral inactivation in rivers in Japan
Source: PLoS One. 2023 Jul 14;18(7):e0288454. doi: 10.1371/journal.pone.0288454 (PMC10348522; doi:10.1371/journal.pone.0288454)
Supplement: S4 Table — (DOCX) [file pone.0288454.s008.docx]

**S4 Table.** MIQE guideline essential information checklist.

| **EXPERIMENTAL DESIGN** |  |  |
| --- | --- | --- |
| Definition of experimental and control groups | **E** | **Y** |
| Number within each group | **E** | **Y** |
| Assay carried out by core lab or investigator's lab? | D | **Y** |
| Acknowledgement of authors' contributions | D | **Y** |
| **SAMPLE** |  |  |
| Description | **E** | **Y** |
| Volume/mass of sample processed | D | **Y** |
| Microdissection or macrodissection | **E** | **NA** |
| Processing procedure | **E** | **Y** |
| If frozen - how and how quickly? | **E** | **NA** |
| If fixed - with what, how quickly? | **E** | **NA** |
| Sample storage conditions and duration (especially for FFPE samples) | **E** | **Y** |
| **NUCLEIC ACID EXTRACTION** |  |  |
| Procedure and/or instrumentation | **E** | **Y** |
| Name of kit and details of any modifications | **E** | **Y** |
| Source of additional reagents used | D | **Y** |
| Details of DNase or RNAse treatment | **E** | **Y** |
| Contamination assessment (DNA or RNA) | **E** | **Y** |
| Nucleic acid quantification | **E** | **Y** |
| Instrument and method | **E** | **Y** |
| Purity (A260/A280) | D | **N** |
| Yield | D | **N** |
| RNA integrity method/instrument | **E** | **N** |
| RIN/RQI or Cq of 3' and 5' transcripts | **E** | **N** |
| Electrophoresis traces | D | **N** |
| Inhibition testing (Cq dilutions, spike or other) | **E** | **Y** |
| **REVERSE TRANSCRIPTION** |  |  |
| Complete reaction conditions | **E** | **Y** |
| Amount of RNA and reaction volume | **E** | **Reaction volume** |
| Priming oligonucleotide (if using GSP) and concentration | **E** | **Y** |
| Reverse transcriptase and concentration | **E** | **Y** |
| Temperature and time | **E** | **Y** |
| Manufacturer of reagents and catalogue numbers | D | **Manufacturer of reagents** |
| Cqs with and without RT | D* | **N** |
| Storage conditions of cDNA | D | **Y** |
| **qPCR TARGET INFORMATION** |  |  |
| If multiplex, efficiency and LOD of each assay. | **E** | **NA** |
| Sequence accession number | **E** | **Please refer to original sources** |
| Location of amplicon | D | **Please refer to original sources** |
| Amplicon length | **E** | **Please refer to original sources** |
| *In silico* specificity screen (BLAST, etc) | **E** | **Please refer to original sources** |
| Pseudogenes, retropseudogenes or other homologs? | D | **N** |
| Sequence alignment | D | **N** |
| Secondary structure analysis of amplicon | D | **N** |
| Location of each primer by exon or intron (if applicable) | **E** | **NA** |
| What splice variants are targeted? | **E** | **NA** |
| **qPCR OLIGONUCLEOTIDES** |  |  |
| Primer sequences | **E** | **Y** |
| RTPrimerDB Identification Number | D | **NA** |
| Probe sequences | D** | **Y** |
| Location and identity of any modifications | **E** | **NA** |
| Manufacturer of oligonucleotides | D | **NA** |
| Purification method | D | **N** |
| **qPCR PROTOCOL** |  |  |
| Complete reaction conditions | **E** | **Y** |
| Reaction volume and amount of cDNA/DNA | **E** | **Y** |
| Primer, (probe), Mg++ and dNTP concentrations | **E** | **Y** |
| Polymerase identity and concentration | **E** | **Y** |
| Buffer/kit identity and manufacturer | **E** | **Y** |
| Exact chemical constitution of the buffer | D | **N** |
| Additives (SYBR Green I, DMSO, etc.) | **E** | **NA** |
| Manufacturer of plates/tubes and catalog number | D | **N** |
| Complete thermocycling parameters | **E** | **Y** |
| Reaction setup (manual/robotic) | D | **NA** |
| Manufacturer of qPCR instrument | **E** | **Y** |
| **qPCR VALIDATION** |  |  |
| Evidence of optimisation (from gradients) | D | **N** |
| Specificity (gel, sequence, melt, or digest) | **E** | **N** |
| For SYBR Green I, Cq of the NTC | **E** | **NA** |
| Standard curves with slope and y-intercept | **E** | **N** |
| PCR efficiency calculated from slope | **E** | **N** |
| Confidence interval for PCR efficiency or standard error | D | **N** |
| r2 of standard curve | **E** | **N** |
| Linear dynamic range | **E** | **N** |
| Cq variation at lower limit | **E** | **N** |
| Confidence intervals throughout range | D | **N** |
| Evidence for limit of detection | **E** | **N** |
| If multiplex, efficiency and LOD of each assay. | **E** | **N** |
| **DATA ANALYSIS** |  |  |
| qPCR analysis program (source, version) | **E** | **qPCR soft 4.1 (analytik jena)** |
| Cq method determination | **E** | **Threshold: 5, Baseline: 5** |
| Outlier identification and disposition | **E** | **N** |
| Results of NTCs | **E** | **Y** |
| Justification of number and choice of reference genes | **E** | **NA** |
| Description of normalisation method | **E** | **NA** |
| Number and concordance of biological replicates | D | **N** |
| Number and stage (RT or qPCR) of technical replicates | **E** | **Duplicate** |
| Repeatability (intra-assay variation) | E | **N** |
| Reproducibility (inter-assay variation, %CV) | D | **N** |
| Power analysis | D | **N** |
| Statistical methods for result significance | **E** | **Y** |
| Software (source, version) | E | **qPCR soft 4.1 (analytik jena)** |
| Cq or raw data submission using RDML | **D** | **N** |
